# Identification of hub genes and therapeutic siRNAs to develop novel adjunctive therapy for Duchenne muscular dystrophy

**DOI:** 10.1186/s12891-024-07206-6

**Published:** 2024-05-18

**Authors:** Na Li, Zhikai Xiahou, Zhuo Li, Zilian Zhang, Yafeng Song, Yongchun Wang

**Affiliations:** 1 Department of Aerospace Medical Training, School of Aerospace Medicine, Air Force Medical University, Xi’an, China; 2 School of Sports Science, Beijing Sport University, Beijing, China; 3 China Institute of Sport and Health Science, Beijing Sport University, Beijing, China

**Keywords:** Duchenne muscular dystrophy, Bioinformatics analysis, Hub genes, siRNAs

## Abstract

**Objective:**

Duchenne muscular dystrophy (DMD) is a devastating X-linked neuromuscular disorder caused by various defects in the dystrophin gene and still no universal therapy. This study aims to identify the hub genes unrelated to excessive immune response but responsible for DMD progression and explore therapeutic siRNAs, thereby providing a novel treatment.

**Methods:**

Top ten hub genes for DMD were identified from GSE38417 dataset by using GEO2R and PPI networks based on Cytoscape analysis. The hub genes unrelated to excessive immune response were identified by GeneCards, and their expression was further verified in *mdx* and C57 mice at 2 and 4 months (M) by (RT-q) PCR and western blotting. Therapeutic siRNAs were deemed as those that could normalize the expression of the validated hub genes in transfected C2C12 cells.

**Results:**

855 up-regulated and 324 down-regulated DEGs were screened from GSE38417 dataset. Five of the top 10 hub genes were considered as the candidate genes unrelated to excessive immune response, and three of these candidates were consistently and significantly up-regulated in *mdx* mice at 2 M and 4 M when compared with age-matched C57 mice, including *Col1a2, Fbn1* and *Fn1*. Furthermore, the three validated up-regulated candidate genes can be significantly down-regulated by three rational designed siRNA (*p* < 0.0001), respectively.

**Conclusion:**

*COL1A2*, *FBN1* and *FN1* may be novel biomarkers for DMD, and the siRNAs designed in our study were help to develop adjunctive therapy for Duchenne muscular dystrophy.

**Supplementary Information:**

The online version contains supplementary material available at 10.1186/s12891-024-07206-6.

## Introduction

Duchenne muscular dystrophy (DMD) is the most frequent hereditary childhood myopathy. It affects predominantly males with an incidence estimated to be about 1 in 3500–5000 live male births [1]. DMD is characterized by progressive muscle degeneration and atrophy leading to premature death in patients in late adolescence due to cardiomyopathy and respiratory failure [2]. Most patients usually present clinical symptoms between 3 and 5 years of age and become incapacitated around 12 years old [3]. DMD is an X chromosome-linked mode of inheritance with various mutations in the gene encoding the skeletal muscle protein dystrophin. This heterogeneity of the mutations is a serious obstacle undermining efforts to repair the primary genetic defect in DMD.

While there is currently no cure for DMD, the recommended clinical therapeutic approaches for it can be briefly categorized into two groups: (1) mutation-specific personalized therapies, which aim to restore the endogenous dystrophin expression, and (2) therapies aiming to compensate for the lack of dystrophin [4]. Stop-codon read-through and antisense oligonucleotide-mediated exon skipping seem to be the most promising mutation-specific therapies and have led to the development of several drugs: ataluren for stop-codon read-through, Exondys51 for exon 51 skipping, Vyondys 53 and Viltepso for exon 53 skipping, Amondys 45 for exon 45 skipping [5–7]. However, both stop-codon read-through and antisense oligonucleotide-mediated exon skipping are only applicable to 10% and 55% of all DMD cases [8]. Importantly, discrepancies remain in the safety and pharmacokinetics of ataluren, and global efforts to improve the cellular uptake and duration of the exon-skipping effect have been undertaken [4]^,^ [9, 10].

Glucocorticoid therapy, a therapeutic method aiming to compensate for the lack of dystrophin, is currently the main clinical treatment for DMD, and could slow down the atrophy rate of skeletal muscle by regulating the proportion of T lymphocyte subsets and inhibiting excessive cellular immune response [11]. This therapy delayed the DMD progression and helped to prolong the lifespan, although it was only targeted at a single immune response pathway and was not a complete cure. Notably, this therapy is irrespective of the mutation type and applicable for all DMD patients. For DMD, a fatal disease in youth, it is also worth developing therapies to improve the quality of life and extend the expectancy by intervening in other pathways and genes that are as important as the excessive immune response before a cure is available.

Recently, emerging studies in DMD patients and animal models have indicated that DMD progression is not fully explained by sarcolemma fragility which was attributed to the absence of dystrophin, aberrant expression of many other genes candidated for many other impaired spectrums also play crucial roles in the development of DMD [12, 13]. Among those dysfunction, abnormalities of calcium homeostasis, neuropsychological impairment and bone deformities are the common dystrophic feature [14]. In addition, deficit in myofiber regeneration, potentially due to an exhaustion of satellite cells, has also been proved to be one of the major pathological features of DMD [15]. Importantly, candidate biomarkers in myonecrosis, inflammation and oxidative stress have recently been regarded as therapeutic targets [16, 17]. However, there are still discrepancies in representative candidate genes, and the underlying intervention approach need further validation. siRNA, a very effective intervention, has achieved remarkable results in the treatment of disease. In 1998, the understanding of gene regulation was revolutionized when researchers discovered that the silencing effectors in *Caenorhabditis elegans* were double stranded RNAs [18]. In the following years, siRNAs were successively used in mammalian cells and mice to specifically silence the expression of different genes which strongly proved the potential of siRNA-therapeutics [19, 20]. In 2018, FDA approve the first siRNA therapeutics (Onpattro) or known as ALN-TTR02 for the treatment of Hereditary Transthyretin Amyloidosis (hATTR) [21]. Additional, HSP47 siRNA designed for moderate-to-severe liver fibers was undergoing a phase I clinical trial to evaluate the safety, tolerability, and pharmacokin- etics (PK) of fixed dose in healthy participant in 2018 [22]. In 2021, siRNAs were applied to significantly illustrate the mechanisms of the skeletal ryanodine receptors (RYR) in impaired myogenic differentiation in human dystrophinopathies and therefore demonstrated the potential value of RYR stabilizers as adjunctive therapy [23]. It can be seen from the above experiments that siRNA therapy relies on identifying key genes that play significant roles in pathogenesis. Therefore, it is urgent to determine the key pathways and central genes of DMD.

In this study present here, to investigate hub genes unrelated to immune response of DMD, we set out to analyze the differentially expressed genes (DEGs) between control and DMD patients in the GSE38417 dataset using integrated bioinformatics analyses. This method including Gene Ontology (GO) term analysis, Kyoto Encyclopedia of Genes and Genomes (KEGG) pathway enrichment analysis, protein-protein interaction (PPI) construction, and the identification of hub genes. Subsequently, the expression of hub genes unrelated to immune system was verified at the mouse level by reverse transcription-quantitative (RT-q) PCR and immunoblot analysis (WB). Finally, the verified hub genes were effectively regulated in C2C12 cells by reasonably designed siRNA. Overall, the results provide therapeutic targets and regulatory approach for the development of adjunctive intervention for DMD.

## Materials & methods

### Data preprocessing and screening of DEGs

GSE38417, a dataset of RNA profiles in control and DMD patients, was retrieved from GEO (Gene Expression Omnibus database, https://www.ncbi.nlm.nih.gov/geo/) using “DMD” and “Homo sapiens” as the keywords. Dorsey SG and Ward CW submitted the GSE38417 dataset which was generated on the GPL570 platform (Affymetrix Human Genome U133 Plus 2.0 Array) using the biotinylated cRNA extracted by Trizol. This dataset included 6 control samples and 16 DMD samples. Of the 16 DMD patients, 5 were less than 3 years old (younger DMD) and 11 were between 3 and 8 years old (older DMD). Considering the clinical symptoms of DMD patients usually onset at 3 years old [24], only older DMD samples were enrolled to search the key factors responsible for DMD progression. DEGs between older DMD patients and control samples were recalculated and assessed using the statistical tool of GEO2R (http://www.ncbi.nlm.nih.gov/geo/geo2r/). The Benjamini–Hochberg method and *t*-tests were used with the GEO2R to calculate the false discovery rate and *p*-values, respectively [25]. After GEO2R, RStudio (version 3.5.3) was used to filter repetitive and discrepant genes. |LogFC| > 1.5 and *p*-value < 0.001 were set as cut-off criteria.

### Functional and pathway enrichment analyses of DEGs

GO analysis is a useful method for functional studies of high-throughput genomic or transcriptomic data, whereas KEGG pathway enrichment analysis is generally applied for systematic analyses of gene functions by linking genomic information with higher-order functional information [26, 27]. GO and KEGG pathway enrichment analyses were performed by using WebGestalt, the WEB-based GEne SeT AnaLysis Toolkit (http://www.webgestalt.org/option.php) [28]. Statistical analyses for biological pathway (BP), cellular component (CC), molecular function (MF), and KEGG with false discovery rate ≤ 0.05 were considered significant.

### PPI networks construction, module analysis, and hub gene identification

Protein–protein interactions (PPIs), commonly understood as physical contacts with molecular docking between proteins that occur in a cell or a living organism in vivo, are emerging as an attractive class of molecular targets for treatment [29]. The STRING is a biological database known to predict and construct PPI networks, in which proteins are nodes and interactions are edges [30]. Hubs that are “highly connected” in a PPI networks are more likely to be essential proteins [31]. In our study, online STRING (version 10.5; http://string-db.org/) was performed to construct PPI networks, and the parameters were set at high confidence > 0.7 with nodes combined score ≥ 0.9. Then, a transformed .csv file from the resulting PPI networks (a “.txt” file) was imported into Cytoscape (version 3.7.2) to visualize the PPI networks [32]. The Cytoscape plug-in Molecular Complex Detection (MCODE) was used to explore significant protein functional modules in the PPI networks, where MCODE scores > 5, degree cut-off = 2, node score cut-off = 0.2, Max depth = 100, and k-core = 2 were used as filtering criteria [33]. By combining the results of the 12 methods listed in the Cytoscape plug-in cytoHubba, the top 10 genes were selected as hub genes [34].

### Mouse lines

As DMD is a progressive disease, the quadriceps muscle tissues of 2 months (2 M) and 4 months (4 M) of muscular dystrophy model (*mdx*) mice (C57BL/ 10ScSn-Dmdmdx/J) and age-match WT mice (C57BL/10ScSn) were extracted to validate the expression of the hub genes. The experiments were executed after animals were deeply anesthetized with isoflurane. There were three *mdx* and C57 mice in each age group. Both strains were purchased from the Jackson Laboratory (Bar Harbor, ME, USA; stock #001801).

### RT-qPCR assay and statistical analysis

Total RNA was extracted by SV Total RNA Isolation System (Promega, Z3100), and at least 500 ng of RNA was used for reverse transcription using the TransScript® Uni One-Step gDNA Removal and cDNA Synthesis SuperMix (Transgen, AU311) following the manufacturer’s instructions. RT-q PCR was performed using PerfectStart® Green qPCR SuperMix(+ Dye I/+Dye II) (Transgen,AQ602) with specific primers for genes (Supplementary Table 1). The 2^−△△Ct^ values were calculated and presented as fold change in gene expression relative to the control group. Vinculin was used as an endogenous control. All of the data were showed as mean ± standard error of mean (SEM) and analyzed using the Prism 9 software. Two-tailed Students t’test was employed to compare between the two groups. *p* < 0.001 was considered statistically significant.

### Immunoblot analysis

WB was carried out by loading 20–40 µg per lane of quadriceps muscle tissues lysate on 4-20% SurePAGETM, Bis-Tris gel(Genscript, M00656). Protein was transferred to a polyvinylidene difluoride membrane. The membrane was blocked in 5% nonfat milk for 1 h at room temperature and incubated with primary antibodies overnight at 4°C: anti-Fibrillin 1 (1:200; Abcom, ab53076), anti-Fibtonectin (1:200; Santa, sc-8422), anti-FYN (1:1000; Abcom, ab125016), anti-COL1A2 (1:200; Santa, sc393573), anti-PKAC-β (1:5000; Abcom, ab76238), and anti-Vinculin (1:10000; Sigma-Aldrich, V9131). The membrane was then incubated with a goat anti-mouse antibody conjugated with horseradish peroxidase (1:10000; ThermoFisher, 31,430) for 1 h at room temperature. Protein detection and quantification were performed using an HRP chemiluminescence detection reagent (ECL, Bio-Rad), and blots were imaged using a ChemiDoc MP imaging system (Bio-Rad). Protein expression was calculated using “target protein/internal reference”, that is, quadriceps muscle target protein (Fibrillin 1, Fibtonectin, FYN, COL1A2, PKAC-β)/ internal reference Vinculin.

### Transient siRNA transfection

C2C12 cells were seeded at 2 × 10^5^ cells/well in Matrigel-coated six-well plates adding 2 ml HG-DMEM and incubated overnight at 37 °C with 5% CO2. The next day, LipofectAMINE 3000 (Thermo Fisher, Paisley, UK) and 10 nM relevant siRNAs (Supplementary Table 2) were pre-diluted in OptiMEM (Thermo Fisher, Paisley, UK) and mixed to form complexes at 37°C for 15 min before being added to cells. *Col1a2*-siRNAs was designed to target the exon 12, *Fbn1*-siRNA was designed to target the exon 42, *Fn1*-siRNA was designed to target exon 11 (Supplementary Table 2). SiRNA transfection was performed 24 h after plating. Total RNA was extracted 24 h after transfection according to the RT-qPCR assay.

## Results

### Identification of DEGs between DMD and control samples in GSE38417 dataset

**Fig. 1:**
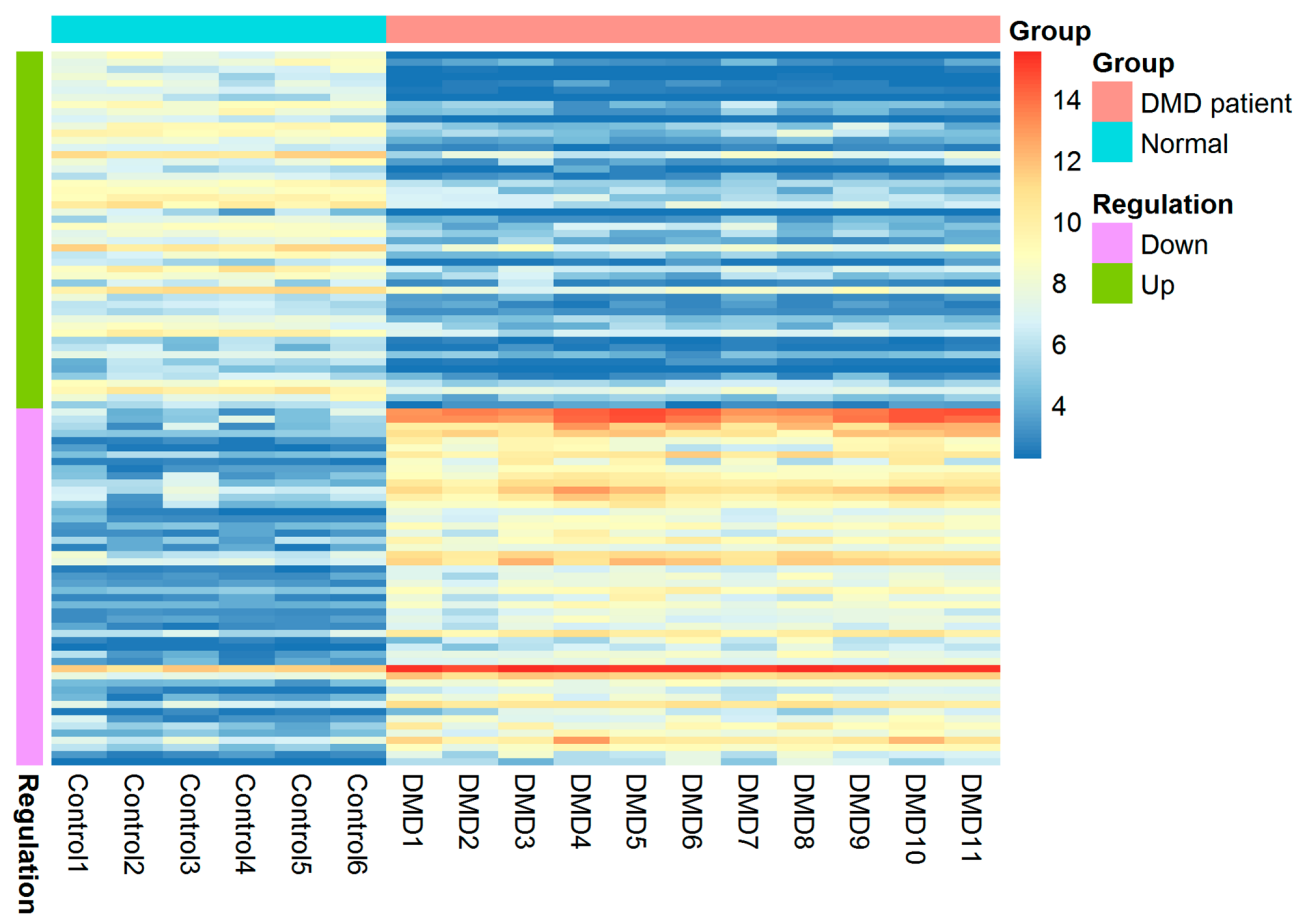
Heatmap of top 100 DEGs of the GSE38417 dataset (50 up-regulated and 50 down-regulated)

In the GSE38417 dataset, 13,843 genes and 1179 DEGs were identified in DMD patients when compared to healthy controls. Among the DEGs, 855 genes were up-regulated, while 324 genes were down-regulated. The expression heatmap of the top 50 up- and down-regulated genes are shown in Fig. 1 and Supplementary Table 3.

.

### Integrative bioinformatics analysis for DEGs screened from GSE38417 dataset

**Fig. 2:**
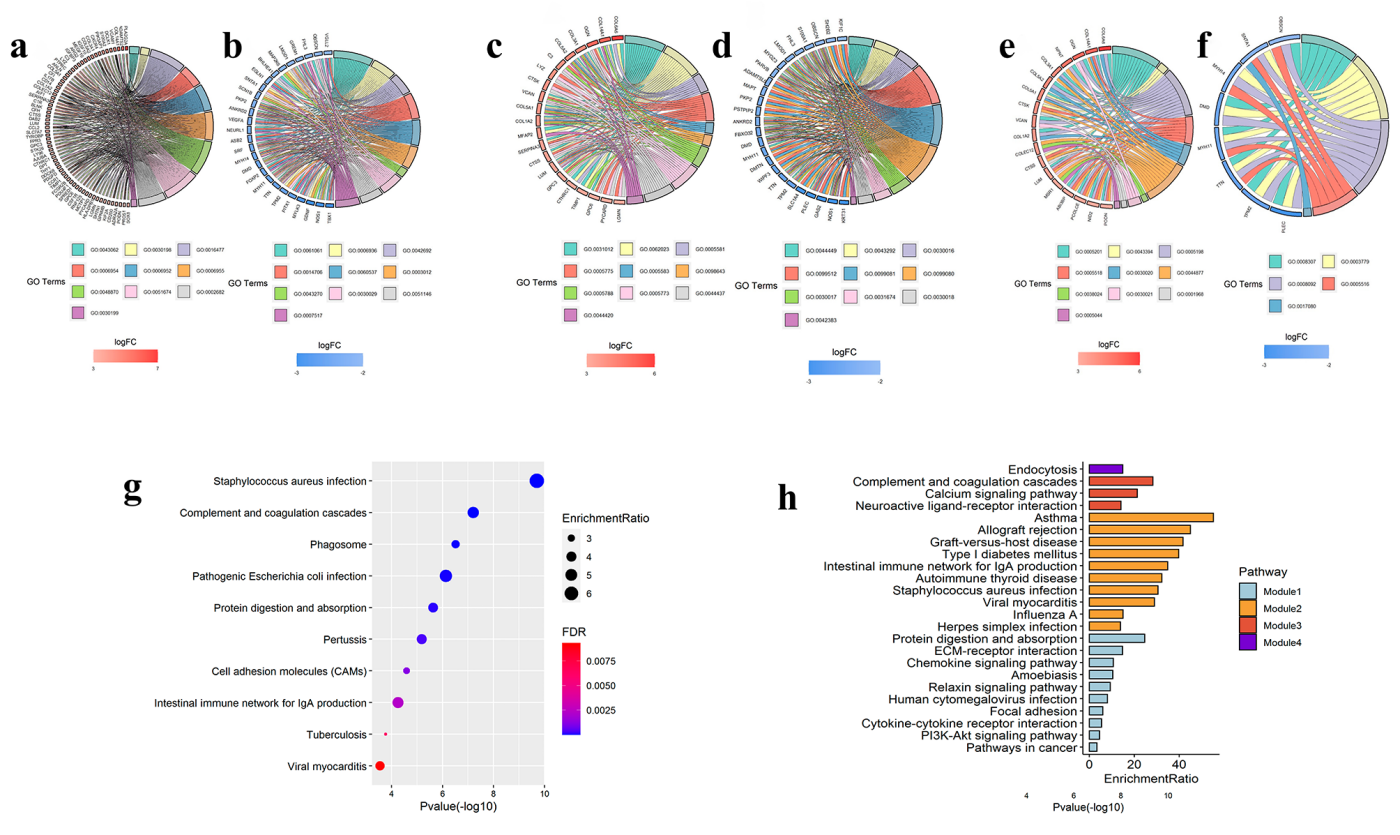
The enrichment analysis of GSE38417 dataset. **a** The top 10 significant functions in the BP category enriched by the top 200 up-regulated DEGs. **b** The top 10 significant functions in the BP category enriched by the top 200 down-regulated DEGs. **c** The top 10 significant functions in the CC category enriched by the top 200 up-regulated DEGs. **d** The top 10 significant functions in the CC category enriched by the top 200 down-regulated DEGs. **e** The top 10 significant functions in the MF category enriched by the top 200 up-regulated DEGs. **f** The top 10 significant functions in the in the MF category enriched by the top 200 down-regulated DEGs. **g** The top 10 items of KEGG pathway enrichment analyses of up-DEGs. The x-axis shows the Pvalue (-log10) of each term, and y-axis shows the KEGG pathway terms. EnrichmentRatio represent the number of enriched genes. **h** Bar plots of enriched pathways of the top four modules screened form PPI networks constructed by the up-regulated DEGs. The x-axis represents the number of enriched genes, the y-axis represents KEGG pathway terms

After the up- and down-regulated DEGs were identified, GO enrichment analysis was performed. The enrichment results of the BP category revealed that up-regulated DEGs were significantly enriched in genes involved in immune response, extracellular matrix (ECM) organization, cell migration, and adhesion (Supplementary Table 4, Fig. 2a); the down-regulated DEGs were enriched in genes involved in muscle structure development, actin filament-based processes, and the positive regulation of ion transport (Supplementary Table 4, Fig. 2b). For the CC category, up-regulated DEGs were associated with the cell surface, ECM, lysosomes, cytoplasmic vesicle particles, and whole membrane (Supplementary Table 4, Fig. 2c), whereas down-regulated DEGs were mostly enriched in parts of contractile fibers, such as I band, sarcomeres, supramolecular complex, and Z discs (Supplementary Table 4, Fig. 2d). Concerning the MF category, up-regulated DEGs were mainly enriched in ECM structural constituents, protein-containing complex binding, and receptor binding (Supplementary Table 4, Fig. 2e), while the down-regulated DEGs were enriched in structural constituents of muscle, actin binding, and cytoskeletal protein binding (Supplementary Table 4, Fig. 2f). In addition, the up-regulated DEGs were significantly enriched in *Staphylococcus aureus* and *Bordetella pertussis* infections, complement and coagulation cascades, phagosomes, and protein digestion and absorption (Fig. 2g), while the down-regulated DEGs did not have any significant KEGG results.

### Construction of the PPI networks of GSE38417 dataset

Considering that the down-regulated DEGs have not enrich any significant KEGG, we only construte the PPI networks of the up-regulated genes. Analysis of the relationship between the 543 nodes and 1849 edges by the MCODE plug-in enabled 4 modules to be selected. Next, a KEGG pathway analysis of genes from these modules was conducted by WebGestalt (Supplementary Table 5, Fig. 2h). The genes involved in module one were mainly involved in protein digestion and absorption, ECM-receptor interactions, and chemokine signaling. The genes in module two were associated with asthma, allograft rejection, type I diabetes mellitus, intestinal immune system network for IgA production, and six other pathways. Moreover, the genes in module three were related to complement and coagulation cascades, calcium signaling pathway, and neuroactive ligand-receptor interactions, while the genes in module four were involved in endocytosis.

### Five hub genes screened from GSE38417 dataset were deemed as candidate hub genes for selecting the novel biomarkers for DMD

According to the information from STRING, the top 10 hub nodes were selected, including fibronectin 1 (*FN1*), complement 3 (*C3*), C-X-C motif chemokine ligand 12 (*CXCL12*), complement component 3a receptor 1 (*C3AR1*), G protein subunit beta 4 (*GNB4*), annexin A1 (*ANXA1*), FYN proto-oncogene of the Src family of tyrosine kinases (*FYN*), fibrillin 1 (*FBN1*), protein kinase cAMP-activated catalytic subunit beta (*PRKACB*), and collagen type I alpha 2 chain (*COL1A2*). GeneCards (https://www.genecards.org/) analysis revealed that *C3, CXCL12, C3AR1, GNB4*, and *ANXA1* were mainly related to the immune system. Considering that glucocorticoid therapy, which aims to compensate for the lack of dystrophin by restricting an excessive immune response, the remaining five hub genes were deemed as the candidate hub genes for selecting the novel biomarkers and therefore only the remaining five hub genes were analyzed in our follow experiments.

### Three up-regulated genes were determined as novel biomarkers for DMD

**Fig. 3:**
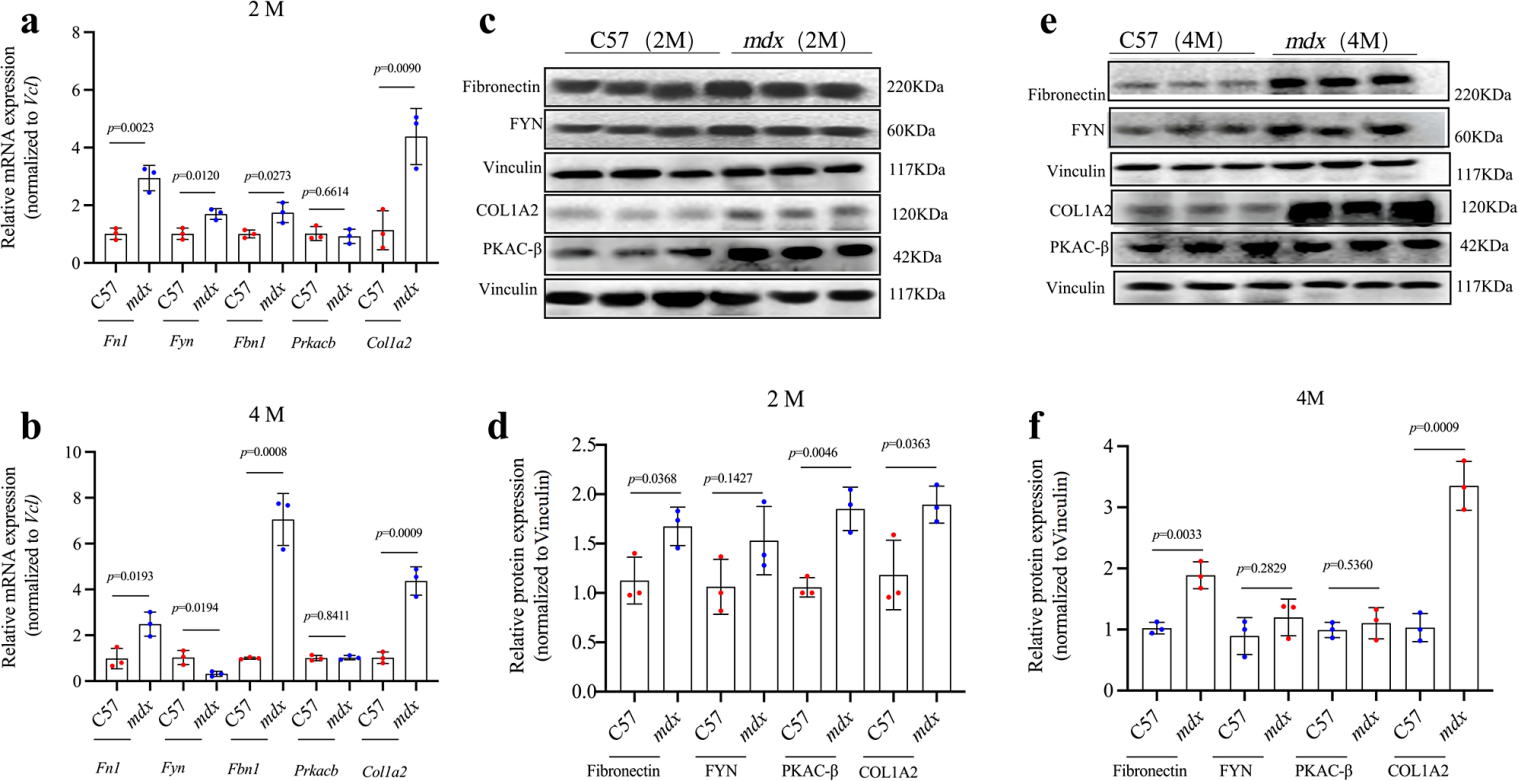
Relative expression levels of *Fn1, Fyn, Fbn1, Col1a2*, and *Prkacb* in quadriceps muscle tissues of C57 and *mdx* mice at 2 M and 4 M. **a** The relative mRNA expression of the 5 genes assessed at 2 M. **b** The relative mRNA expression of the 5 genes assessed at 4 M. **c d** The relative protein expression of *Col1a2, Fyn, Prkacb*, and *Fn1* assessed at 2 M. **e f** The relative protein expression of *Col1a2, Fyn, Prkacb*, and *Fn1* assessed at 4 M. *n* = 3 biologically independent samples. Wild-type (C57) expression levels were set at 1. Data are mean ± standard deviation, and a two-tailed Student’s *t*-test was used to calculate *p*-values.The grouping of blots cropped from different gels, each blot was divided with black lines. Since the blots were cut prior to hybridization with the antibody, the original image of the full-length bolts cannot be showed here, but images with the visible membrane edge were provided in the Supplementary Information file

To confirm the involvement of *FN1*, *FYN, FBN1, PRKACB and COL1A2* in DMD progression, the levels of mRNA and protein expression of *Fn1*, *Fyn*, *Fbn1*, *Prkacb* and *Col1a2* in *mdx* and C57 mice at 2 and 4 months (M) of age were measured by using (RT-q) PCR and WB analysis. *Fn1* (*p* = 0.0023 at 2 M, *p* = 0.0193 at 4 M), *Fbn1* (*p* = 0.0273 at 2M, *p* = 0.0008 at 4 M) and *Col1a2* (*p* = 0.0090 at 2 M, *p* = 0.0009 at 4 M) mRNA was significantly up-regulated in *mdx* mice both at 2 and 4 M. However, *Prkacb* mRNA was similar in *mdx* and C57 mice at 2 and 4 M, *Fyn* was up-regulated in *mdx* mice at 2 M but similar with C57 mice at 4 M (Fig. 3a.b). WB analysis indicated that the relative protein expression of Fibtonectin (*p* = 0.0368 at 2 M, *p* = 0.0033 at 4 M), and COL1A2 (*p* = 0.0363 at 2 M, *p* = 0.0009 at 4 M) were consistent with the results of bioinformatics and (RT-q)PCR. Just like the results of RT-qPCR, the protein levels of FYN and PKAC-β were not persistent over-expression in *mdx* mice at the time points in this study(Figs. 1S and 3c, d, e and f). However, there were a failure in detection the protein level of fibrillin 1, the only obtained primary antibodies not suitable for WB maybe the reason. Therefore, in addition to *Col1a2* and *FN1* being determined as novel biomarkers for DMD, *FBN1* was still selected as novel biomarker for DMD, the conclusion were based on the results of bioinformatics, (RT-q)PCR and the consistency of the results of (RT-q)PCR and WB in *FN1* and *Col1a2.*

### The gene-targeted siRNAs that normalize the expression of novel biomarkers were considered a new approach to treat DMD

**Fig. 4:**
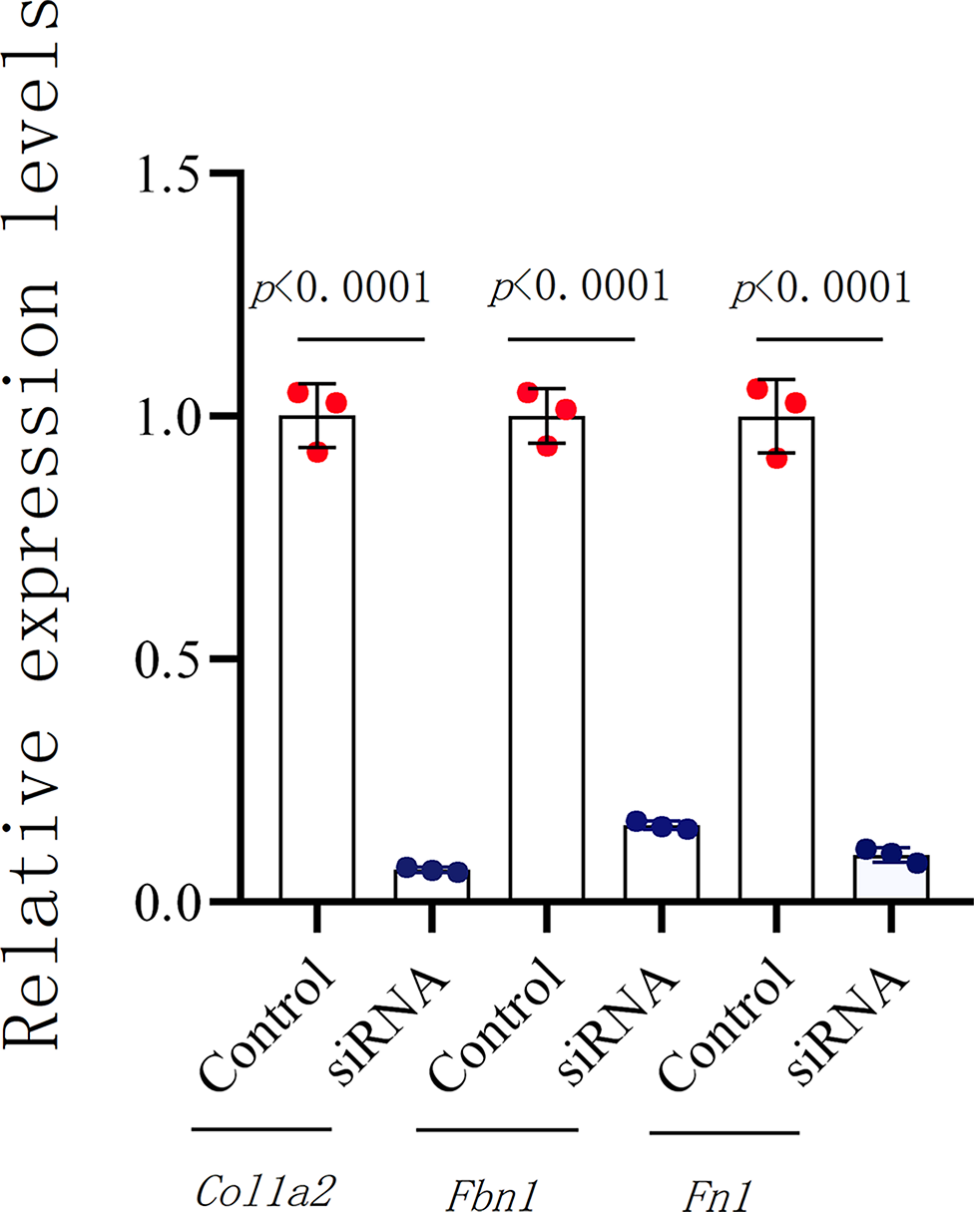
Relative expression of *Col1a2*, *Fbn1*, and *Fn1* in C2C12 were assessed by (RT-q) PCR. C2C12 cells at 80% of confluence were incubated with scramble or gene-specific siRNAs. Scramble siRNAs expression levels were set at 1. Data are mean ± standard deviation, and a two-tailed Student’s *t*-test was used to calculate *p*-values

The three validated biomarkers are all up-regulated in DMD patients and *mdx* mice, which means that normalized the expression level could be beneficial for the treatment of DMD. By transfecting three rational designed gene-targets siRNAs to C2C12 cells, the expression of the validated novel biomarkers were significantly decreased in transfected C2C12 cells, with *Col1a2* (*p* < 0.0001), *Fbn1* (*p* < 0.0001) and *Fn1* (*p* < 0.0001), respectively (Fig. 4).

## Discussion

DMD is a disease characterized by severe, progressive muscle degeneration associated ultimately with cardiac and pulmonary dysfunction [35]. Our study revealed 1179 up-regulated DEGs that are mainly enriched in the immune response, ECM structure-associated activity, and viral myocarditis, as well as 324 down-regulated DEGs that are mainly enriched in muscle structure, regulation, and function. These results are consistent with the notion that the immune system plays an important role in dystrophic muscle disease pathogenesis, sustaining continuous repetitive cycles of inflammatory and fibrotic responses [36, 37].

KEGG analysis of the top four modules in the PPI networks of up-regulated DEGs showed that ECM-receptor interaction, chemokine signaling pathways, complement and coagulation cascades, and calcium signaling pathways were dysfunctional in DMD. This is in line with previous studies that demonstrated that immune cell infiltration of the muscles in *mdx* mice and transforming growth factor-β (TGF-β)-mediated inflammation could cause the progressive deposition of fibrous ECM [38]. Moreover, chronic damage and inflammation in DMD has been shown to induce elevated TGF-β activity, which allows fibroadipogenic progenitors to differentiate into fibrogenic and other ECM-secreting cells thus leading to muscle fiber calcification [39]. Encouragingly, a recent study revealed that regulating TGF-β1/Smad3 signaling by the coreceptor for TGF-β receptor type II (TβR II) could reduce muscle-wasting [40]. Similarly, calcium homeostasis in myoblasts was altered profoundly by the mutant *Dmd* gene [41].

In the current study, however, CXCL12 was screened as hub nodes but not deemed as target hub gene due to the involvement of immune response, but this result was highly consistent with another study which was also based on the GSE 38,417 dataset [42]. Lai et al. revealed that CXCL12 was a glucocorticoid targeted DEG and thereby a potential therapeutic target in DMD. Among the five hub nodes which were not associated with the immune response, *COL1A2,FN1* and *FBN1*,were significantly up-regulated in older DMD patients analyzed by bioinformatics and *mdx* mice detected or calculated by RT-qPCR and WB. *COL1A2* encodes the alpha chain of type I collagen, and whose significantly higher expression in DMD than controls has been indicated by the previous study which further determined that the alpha chain of type I collagen accumulation is responsible for the skeletal muscle fibrosis in DMD [43]. As the result of our study, over-expression of *FBN1* induced DMD, however, patients with Marfan syndrome (MFS), which is caused by an *FBN1* mutation as well as *Fbn1*-deficient mice present some phenotypes similar to DMD, such as a decrease in the size and number of myofibers accompanied by an increase in fragmented fibers [44–47]. An additional study demonstrated that FBN1, which is a crucial component of connective tissue elastic fibers and an important extracellular regulator of TGF-β activity, could be linked to muscle atrophy and impaired muscle regeneration. Therefore, *FBN1* may have a significant supporting effect on maintaining the structure and function of muscle, and both low- and over-expression of *FBN1* could induce muscle dysfunction [48, 49]. Moreover, over-expression of the extracellular matrix glycoprotein FN1 was also detected in our experimental data. This is highly consistent with previous studies that revealed fibronectin is a serum biomarker for Duchenne muscular dystrophy [50].Another study also revealed that up-regulation of *FN1* induced the deposition of fibronectin in the cytoplasm, which causes fibrosis [51]. Finally, other studies demonstrated that activated fibroblasts proliferate and express high levels of extracellular proteins, which leads to the expansion of fibrotic tissue [52].

The lack of significantly high expression of *Fyn* and *Prkacb* in *mdx* mice may be attributed to the milder phenotypes that these mutations cause as compared with that seen in DMD patients. FYN is a member of the Src family of nonreceptor tyrosine kinases that plays a role in many biological processes including regulation of cell growth and survival, integrin-mediated signaling, cytoskeletal remodeling, and cell motility. One of the mechanisms of up-regulated *FYN*, which could account for the DMD phenotype is that the Fyn-tyrosine kinase activates the mammalian target of rapamycin 1 (mTORC1) signaling complex, which inhibits macroautophagy and induces marked muscular atrophy [53]. *PRKACB* is another gene that plays an important role in cardiac and skeletal muscles. Several studies have asserted that upon equal stimulation, myocytes exhibit stronger contractions in the presence of β-agonists because of the induced increase in the levels of cAMP [54, 55]. Furthermore, treatment with β-agonists up-regulated *PRKACB* when compared with controls [56]. Therefore, the high expression level of *PRKACB* in DMD, which is similar to up-regulated *PRKACB* upon β-agonist treatment, is probably a result of genetic compensatory response to the muscle degeneration in DMD. However, a recent study has illustrated that *PRKACB* has a close relationship with immune cells, especially M2 macrophages [57]. Therefore, the regulation mechanism of PRKACB requires further study.

siRNAs have been shown to play important roles in gene regulation that impact various diseases. In the final content of our study, rational siRNAs targeted to the coding sequence of three up-regulated hub genes were synthesized according to design principals [58], and transfected into C2C12 cell to regulate the expression of the targeted genes. RT-qPCR indicated that those siRNAs could significantly down-regulate the mRNA expression of the target gene. This results further suggested the therapeutic potential of these siRNAs. However, how to safely and efficiently deliver siRNA drugs to specific target cells and protect them from degradation is one of the major obstacles of current siRNA therapy. Lipid nanoparticles (LNP) are the most advanced siRNA delivery vectors in clinical practice. However, clinical studies have shown that LNP accumulates in the liver, so current LNP delivery systems are mostly liver targeted, and effective delivery of LNP in muscle needs to be addressed urgently. In a recent study, a selective organ targeting lipid nanoparticles named SORT (selective organ targeting) were developed to specifically target liver, lung, spleen and other organs by adding a new lipid SORT lipid [59]. Meanwhile, the specific mechanism of tissue-specific delivery of selective organ targeted lipid nanoparticles has also been clarified. They believe that adjusting the molecular composition of nanoparticles to bind to specific proteins in serum can be delivered to the target site. This may be an effective strategy for developing the muscle target nanocarriers, and help to deliver the siRNA-therapeutics to DMD patients to mitigate the DMD progress.

In summary, *COL1A2*, *FBN1*, and *FN1* were hub genes irrespective of immune response but responsible for DMD progression. The siRNAs designed in our study were help to develop adjunctive therapy for Duchenne muscular dystrophy.

### Electronic supplementary material

Below is the link to the electronic supplementary material.

Supplementary Material 1

Supplementary Material 2

Supplementary Material 3

Supplementary Material 4

Supplementary Material 5

Supplementary Material 6

## Data Availability

The GSE38417 dataset used and/or analysed during the current study available from GEO.
