# Supplementary material for: Identification of hub genes and therapeutic siRNAs to develop novel adjunctive therapy for Duchenne muscular dystrophy
Source: BMC Musculoskelet Disord. 2024 May 18;25:386. doi: 10.1186/s12891-024-07206-6 (PMC11102231; doi:10.1186/s12891-024-07206-6)
Supplement: Supplementary file 1 — Supplementary Material 1 [file 12891_2024_7206_MOESM1_ESM.docx]

**Supplementary table 1. Primers and their sequences for (RT-q)PCR analysis.**

| Primer | Sequence (5′–3′) |
| --- | --- |
| Vcl-F | TTACAGTGGCAGAGGTAGTGGAA |
| Vcl-R | TGAAATGAGAACAGGAAGCAGC |
| Col1a2-F | CTCCCGGTATTCTGGGTCTC |
| Col1a2-R | AGGGTTACCATCACGACCAG |
| Fyn-F | AGTTGCCAAACCTTGTGGAC |
| Fyn-R | CCGAGCCAATCCAAAGTCAG |
| Fbn1-F | ATGAGTGCAGCATCCGAAAC |
| Fbn1-R | CATCTGTAGGAGCCATCCGT |
| Prkacb-F | AAAGGTCCGGTTCCCATCA |
| Prkacb-R | ACTCACGCCGTTCTTCAGGT |
| Fn1-F | TATTTACCAACCGCAGACTCAC |
| Fn1-R | GCTTGTTTCCTTGCGACTTC |
