# Supplementary material for: Identification of hub genes and therapeutic siRNAs to develop novel adjunctive therapy for Duchenne muscular dystrophy
Source: BMC Musculoskelet Disord. 2024 May 18;25:386. doi: 10.1186/s12891-024-07206-6 (PMC11102231; doi:10.1186/s12891-024-07206-6)
Supplement: Supplementary file 2 — Supplementary Material 2 [file 12891_2024_7206_MOESM2_ESM.docx]

**Supplementary table 2. siRNAs sequences for each validated hub genes.**

| Gene | Sense Sequence (5′–3′) |
| --- | --- |
| *Col1a2*-siRNA | GACACAGTGGTATGGATGGAT |
| *Fbn1*-siRNA | CCGGCTTCGTGATTGACATTT |
| *Fn1*-siRNA | GTGCATCGTTGATGACATTAC |
