# Supplementary material for: Identification of hub genes and therapeutic siRNAs to develop novel adjunctive therapy for Duchenne muscular dystrophy
Source: BMC Musculoskelet Disord. 2024 May 18;25:386. doi: 10.1186/s12891-024-07206-6 (PMC11102231; doi:10.1186/s12891-024-07206-6)
Supplement: Supplementary file 3 — Supplementary Material 3 [file 12891_2024_7206_MOESM3_ESM.docx]

**Supplementary table 3. DEGs between DMD and healthy controls in GSE38417**

| Gene.symbol | adj.P.Val | logFC | Disease |
| --- | --- | --- | --- |
| COL3A1 | 4.06E-10 | 3.89 | Up |
| NIPSNAP3A | 6.45E-10 | 2.39 | Up |
| COL5A2 | 9.44E-10 | 3.89 | Up |
| COL1A2 | 9.44E-10 | 3.4 | Up |
| SPARC | 9.44E-10 | 2.77 | Up |
| FZD10 | 1.16E-09 | 4.3 | Up |
| TP53I3 | 1.16E-09 | 3.62 | Up |
| CRISPLD1 | 1.7E-09 | 4.23 | Up |
| MARCKS | 2.29E-09 | 2.58 | Up |
| COL6A6 | 7.4E-09 | 5.92 | Up |
| FAM198B | 7.4E-09 | 2.4 | Up |
| OSTC | 7.4E-09 | 1.8 | Up |
| PEG3 | 7.94E-09 | 3.38 | Up |
| GNB4 | 7.94E-09 | 2.69 | Up |
| MYH8 | 1.01E-08 | 8.16 | Up |
| MEGF10 | 1.01E-08 | 3.86 | Up |
| COL5A1 | 1.01E-08 | 3.51 | Up |
| ADAMTS2 | 1.16E-08 | 5.03 | Up |
| LRRC17 | 1.58E-08 | 5.5 | Up |
| NRK | 1.58E-08 | 4.32 | Up |
| PLAGL1 | 1.89E-08 | 3.27 | Up |
| GXYLT2 | 1.99E-08 | 3.39 | Up |
| CELF2 | 1.99E-08 | 3.16 | Up |
| ZEB2 | 2.24E-08 | 2.09 | Up |
| DCLK1 | 2.46E-08 | 4.16 | Up |
| ASPN | 2.77E-08 | 2.73 | Up |
| MYL5 | 2.93E-08 | 6.16 | Up |
| LAMB1 | 0.00000003 | 2.71 | Up |
| TUBA1A | 0.00000003 | 2.48 | Up |
| PALM2-AKAP2///AKAP2 | 0.00000003 | 2.27 | Up |
| NBPF20///NBPF10///NBPF8///NBPF9///NBPF15///NBPF12///NBPF14 | 0.00000003 | 1.97 | Up |
| GLT8D2 | 3.02E-08 | 3.09 | Up |
| VCAM1 | 3.58E-08 | 4.46 | Up |
| PLPP5 | 3.79E-08 | 2.79 | Up |
| DMXL2 | 3.99E-08 | 3.14 | Up |
| SPRED1 | 4.02E-08 | 2.95 | Up |
| MS4A6A | 4.67E-08 | 4.23 | Up |
| MYH3 | 4.79E-08 | 8.66 | Up |
| RIPK2 | 5.01E-08 | 2.06 | Up |
| CASP1 | 5.21E-08 | 3.26 | Up |
| ZCCHC7 | 5.25E-08 | 1.74 | Up |
| SPIN4 | 5.38E-08 | 2.63 | Up |
| DOCK8 | 5.41E-08 | 3.04 | Up |
| ELK3 | 5.49E-08 | 2.12 | Up |
| NBPF20///NBPF10///NBPF8///NBPF9///NBPF15///NBPF11///NBPF12///NBPF14 | 5.65E-08 | 1.97 | Up |
| MYO1B | 6.25E-08 | 2.14 | Up |
| COLEC12 | 6.43E-08 | 3.36 | Up |
| COL6A3 | 6.43E-08 | 2.6 | Up |
| PPIP5K2 | 6.43E-08 | 2.13 | Up |
| CCNG2 | 6.57E-08 | 2.37 | Up |
| NBPF10///NBPF8///NBPF11 | 6.57E-08 | 2.15 | Up |
| MEST | 0.000000066 | 3.85 | Up |
| TMSB10 | 6.78E-08 | 1.95 | Up |
| FRMD6 | 6.84E-08 | 3.09 | Up |
| TTC28 | 6.89E-08 | 1.67 | Up |
| VASH2 | 6.92E-08 | 4.56 | Up |
| CFHR1///CFH | 0.00000007 | 2.97 | Up |
| EPB41L3 | 7.24E-08 | 3.9 | Up |
| CTSK | 7.24E-08 | 3.62 | Up |
| SNORD83B///SNORD139///RPL3 | 7.24E-08 | 2.15 | Up |
| CHRNA1 | 7.28E-08 | 4.31 | Up |
| KCTD12 | 7.34E-08 | 1.97 | Up |
| PEG10 | 7.51E-08 | 3.47 | Up |
| CLCN5 | 7.63E-08 | 2.98 | Up |
| DSEL | 7.64E-08 | 3.19 | Up |
| BEX4 | 7.98E-08 | 2.56 | Up |
| FMO1 | 0.000000083 | 4.02 | Up |
| GOLM1 | 0.000000083 | 3.25 | Up |
| CASP4 | 0.000000083 | 2.79 | Up |
| NBPF1 | 0.000000083 | 2.02 | Up |
| NBPF26///NBPF10///NBPF14 | 0.000000083 | 1.92 | Up |
| COL15A1 | 0.000000091 | 2.21 | Up |
| SSR1 | 9.69E-08 | 1.72 | Up |
| RPL3 | 9.71E-08 | 2.08 | Up |
| FKBP11 | 0.000000101 | 2.16 | Up |
| PELI1 | 0.000000101 | 1.79 | Up |
| GPR137B | 0.000000102 | 3.48 | Up |
| LUM | 0.000000109 | 3.15 | Up |
| TCEAL9 | 0.000000111 | 2.22 | Up |
| PLP1 | 0.000000116 | 4.02 | Up |
| FGL2 | 0.000000122 | 3.17 | Up |
| CFI | 0.000000124 | 3.51 | Up |
| HTRA1 | 0.000000131 | 2.76 | Up |
| ZNF738 | 0.000000133 | 2.37 | Up |
| SEPT11 | 0.000000134 | 1.9 | Up |
| RBMS1 | 0.00000014 | 1.79 | Up |
| STK26 | 0.000000145 | 3.1 | Up |
| MIF | 0.00000017 | 1.73 | Up |
| PAG1 | 0.000000173 | 2.26 | Up |
| MPEG1 | 0.000000177 | 3.31 | Up |
| YWHAQ | 0.000000177 | 1.62 | Up |
| TCF7L2 | 0.000000179 | 1.75 | Up |
| TANC2 | 0.000000183 | 3.59 | Up |
| ABI3BP | 0.000000185 | 3.02 | Up |
| SAT1 | 0.000000187 | 2.97 | Up |
| SNORD77///SNORD76///SNORD74///GAS5///SNORD44///SNORD47///SNORD80///SNORD79///SNORD81 | 0.000000187 | 1.94 | Up |
| UBTD2 | 0.000000187 | 1.59 | Up |
| ABRACL | 0.000000188 | 3.15 | Up |
| MXRA5 | 0.000000199 | 4.32 | Up |
| CCDC80 | 0.000000201 | 4.83 | Up |
| TMEM45A | 0.000000201 | 3.51 | Up |
| NBPF25P///NBPF26///NBPF19///NBPF20///NBPF10///NBPF8///NBPF9///NBPF15///NBPF11///NBPF14 | 0.000000207 | 1.86 | Up |
| OGN | 0.000000214 | 4.92 | Up |
| EFHC1 | 0.000000218 | 2.41 | Up |
| IGSF10 | 0.000000221 | 3.86 | Up |
| C1RL | 0.000000226 | 2.36 | Up |
| CMAHP | 0.000000231 | 2.44 | Up |
| MS4A4A | 0.000000239 | 4.01 | Up |
| WWTR1 | 0.000000239 | 2.19 | Up |
| HOXB-AS1 | 0.000000241 | 2.94 | Up |
| MCC | 0.000000241 | 1.93 | Up |
| PLK2 | 0.000000242 | 2.1 | Up |
| PIK3AP1 | 0.000000245 | 3.92 | Up |
| VGLL3 | 0.000000245 | 3.42 | Up |
| ZNF217 | 0.000000253 | 2.27 | Up |
| GIMAP2 | 0.00000027 | 3.04 | Up |
| DPY19L3 | 0.00000027 | 2.2 | Up |
| DAB2 | 0.000000272 | 3.16 | Up |
| CHN1 | 0.000000272 | 2.62 | Up |
| NOTCH2 | 0.000000274 | 2.29 | Up |
| FMR1 | 0.000000274 | 1.97 | Up |
| DAAM1 | 0.00000028 | 1.94 | Up |
| DPT | 0.000000281 | 3.05 | Up |
| FAT1 | 0.000000281 | 2.64 | Up |
| SNAI2 | 0.000000281 | 2.26 | Up |
| ENPP2 | 0.000000287 | 2.68 | Up |
| LGMN | 0.00000029 | 2.86 | Up |
| PTGFRN | 0.000000295 | 2.85 | Up |
| GPM6B | 0.000000306 | 3.48 | Up |
| LPAR1 | 0.000000318 | 2.75 | Up |
| REV3L | 0.000000322 | 1.71 | Up |
| PSD3 | 0.000000323 | 2.34 | Up |
| KIF13B | 0.000000329 | 1.61 | Up |
| ATP11C | 0.000000342 | 1.96 | Up |
| PCDH19 | 0.000000342 | 1.71 | Up |
| SHTN1 | 0.000000346 | 2.86 | Up |
| SESN3 | 0.000000348 | 3.49 | Up |
| SRPX | 0.000000351 | 4 | Up |
| MFHAS1 | 0.000000351 | 1.88 | Up |
| IFI16 | 0.000000352 | 2.74 | Up |
| TEP1 | 0.000000353 | 1.65 | Up |
| OLFML2B | 0.000000354 | 2.81 | Up |
| SGCE | 0.000000356 | 1.93 | Up |
| ZNF521 | 0.000000375 | 2.75 | Up |
| CPQ | 0.000000377 | 1.66 | Up |
| SERINC5 | 0.000000393 | 2.45 | Up |
| SP100 | 0.000000393 | 1.58 | Up |
| SEC24D | 0.000000402 | 2.5 | Up |
| HLA-DRA | 0.000000408 | 2.66 | Up |
| PCOLCE | 0.000000418 | 3 | Up |
| TUBB6 | 0.000000427 | 3.35 | Up |
| MAP3K5 | 0.000000465 | 1.85 | Up |
| PNMAL1 | 0.000000466 | 4.47 | Up |
| MCUB | 0.000000467 | 3.2 | Up |
| C1R | 0.000000467 | 3.2 | Up |
| JAM2 | 0.000000467 | 1.96 | Up |
| RHOJ | 0.000000467 | 1.75 | Up |
| CLIC1 | 0.000000468 | 2.06 | Up |
| CMTM7 | 0.000000469 | 2.62 | Up |
| ETNK1 | 0.000000474 | 1.83 | Up |
| SLC9A9 | 0.000000484 | 2.46 | Up |
| TSPAN6 | 0.000000485 | 1.63 | Up |
| CD163 | 0.000000489 | 4.24 | Up |
| TIMP2 | 0.000000489 | 2.17 | Up |
| HIF1A | 0.000000498 | 1.98 | Up |
| THBS2 | 0.000000513 | 3.7 | Up |
| SFT2D2 | 0.000000529 | 1.7 | Up |
| PTPRC | 0.000000539 | 3.58 | Up |
| NPC2 | 0.000000539 | 1.64 | Up |
| CCDC8 | 0.000000546 | 2.24 | Up |
| MAN1A1 | 0.000000559 | 2.42 | Up |
| LHFPL2 | 0.000000581 | 2.66 | Up |
| NPNT | 0.000000583 | 4.57 | Up |
| LYN | 0.000000586 | 2.14 | Up |
| PODN | 0.000000599 | 2.82 | Up |
| CASP7 | 0.000000633 | 1.65 | Up |
| NID1 | 0.000000638 | 2.38 | Up |
| TSHZ3 | 0.00000064 | 2.33 | Up |
| C3 | 0.000000647 | 3.67 | Up |
| TMEM123 | 0.000000664 | 1.85 | Up |
| ARPC5 | 0.000000674 | 1.96 | Up |
| AMMECR1 | 0.000000683 | 2.93 | Up |
| HSD17B11 | 0.000000688 | 1.66 | Up |
| TYROBP | 0.000000697 | 3.15 | Up |
| GPNMB | 0.0000007 | 2.84 | Up |
| CMTM6 | 0.000000718 | 2.06 | Up |
| FAM60A | 0.000000725 | 1.54 | Up |
| GRK3 | 0.000000741 | 3.28 | Up |
| HLA-DPB1 | 0.000000741 | 2.87 | Up |
| THY1 | 0.000000745 | 3.05 | Up |
| ZSWIM6 | 0.000000753 | 2.3 | Up |
| TUBB2A | 0.000000764 | 1.6 | Up |
| VWA5A | 0.000000773 | 3.03 | Up |
| GLMP | 0.000000774 | 2.3 | Up |
| PROS1 | 0.000000803 | 2.82 | Up |
| ZMAT3 | 0.000000809 | 1.53 | Up |
| TMEM98 | 0.00000081 | 2.92 | Up |
| VCAN | 0.000000819 | 3.58 | Up |
| FAM172A | 0.00000082 | 2.09 | Up |
| ROBO1 | 0.00000082 | 2.08 | Up |
| PSMB8 | 0.000000849 | 2.64 | Up |
| CHRDL1 | 0.00000086 | 3.49 | Up |
| KCTD3 | 0.000000868 | 1.8 | Up |
| RAB8B | 0.000000876 | 1.99 | Up |
| CD200 | 0.00000089 | 2.83 | Up |
| AHR | 0.000000905 | 2.4 | Up |
| NID2 | 0.00000091 | 2.89 | Up |
| CHODL | 0.000000918 | 2.08 | Up |
| STK17B | 0.000000941 | 2.6 | Up |
| MS4A7 | 0.000000956 | 4.33 | Up |
| DNM3OS | 0.000000956 | 3.34 | Up |
| CTHRC1 | 0.00000097 | 3.06 | Up |
| ADAP2 | 0.000000976 | 2.55 | Up |
| RCC2 | 0.000000977 | 1.59 | Up |
| ELAVL2 | 0.000000978 | 3.26 | Up |
| POU4F1 | 0.000000981 | 2.97 | Up |
| CLIC4 | 0.000000981 | 2.22 | Up |
| C19orf66 | 0.000000987 | 1.99 | Up |
| CD86 | 0.000000992 | 3 | Up |
| MYOF | 0.000000992 | 2.97 | Up |
| LY75-CD302///CD302///LY75 | 0.000000992 | 2.47 | Up |
| CTSS | 0.000000993 | 3.18 | Up |
| SYNE3///LINC00341 | 0.000000993 | 2.41 | Up |
| MFAP2 | 0.000000995 | 3.33 | Up |
| UCP2 | 0.000001 | 1.92 | Up |
| LGALS3BP | 0.00000101 | 2.12 | Up |
| TNFSF13B | 0.00000102 | 3.77 | Up |
| PARP4 | 0.00000102 | 1.71 | Up |
| FAM122B | 0.00000103 | 1.64 | Up |
| LY96 | 0.00000105 | 3.1 | Up |
| HMGN1 | 0.00000105 | 1.72 | Up |
| COL4A1 | 0.00000106 | 1.65 | Up |
| IKBIP | 0.00000107 | 2.28 | Up |
| FAM161A | 0.0000011 | 2.51 | Up |
| STIM2 | 0.0000011 | 1.55 | Up |
| SOX11 | 0.00000112 | 3.06 | Up |
| ADD3 | 0.00000114 | 2.09 | Up |
| CXCL12 | 0.00000115 | 1.91 | Up |
| RARRES3 | 0.00000116 | 2.19 | Up |
| FAM91A1 | 0.00000116 | 1.63 | Up |
| RASSF2 | 0.00000118 | 2.59 | Up |
| RLIM///PABPC1 | 0.00000118 | 1.53 | Up |
| PLA2G2A | 0.00000119 | 6.68 | Up |
| JAM3 | 0.00000119 | 2.2 | Up |
| LAPTM5 | 0.0000012 | 3.29 | Up |
| BEX3 | 0.0000012 | 1.52 | Up |
| AIF1 | 0.00000121 | 3.27 | Up |
| CYS1 | 0.00000124 | 2.35 | Up |
| HPS3 | 0.00000127 | 1.74 | Up |
| SLC44A1 | 0.00000128 | 1.62 | Up |
| FAT4 | 0.00000129 | 2.16 | Up |
| EFS | 0.00000129 | 1.59 | Up |
| BLNK | 0.00000131 | 3.19 | Up |
| SLC38A6 | 0.00000131 | 1.88 | Up |
| FBXL7 | 0.00000131 | 1.63 | Up |
| BTN3A2///BTN3A3 | 0.00000131 | 1.55 | Up |
| HLA-DPA1 | 0.00000132 | 2.59 | Up |
| LGALS8 | 0.00000132 | 1.78 | Up |
| IL33 | 0.00000133 | 2.67 | Up |
| HLA-DRB1 | 0.00000133 | 1.91 | Up |
| BACE2 | 0.00000133 | 1.89 | Up |
| CARD16///CASP1 | 0.00000134 | 2.33 | Up |
| SLC2A10 | 0.00000135 | 3.23 | Up |
| PYGL | 0.00000137 | 2.4 | Up |
| TANC1 | 0.00000137 | 1.67 | Up |
| ELF4 | 0.00000139 | 1.78 | Up |
| KIF2A | 0.00000141 | 2.84 | Up |
| RNASE6 | 0.00000143 | 3.51 | Up |
| RGL1 | 0.00000145 | 3.37 | Up |
| BTG1 | 0.00000145 | 1.77 | Up |
| GPX8 | 0.00000148 | 3.09 | Up |
| TNFRSF21 | 0.00000149 | 2.38 | Up |
| SH3BGRL | 0.00000151 | 2.18 | Up |
| PREPL | 0.00000154 | 1.95 | Up |
| DTX3L | 0.00000155 | 1.85 | Up |
| IFT52 | 0.00000156 | 1.73 | Up |
| CCND2 | 0.00000157 | 3.28 | Up |
| CILP | 0.00000157 | 2.8 | Up |
| COL5A3 | 0.00000157 | 1.87 | Up |
| RNF125 | 0.00000158 | 2.91 | Up |
| ARL15 | 0.00000158 | 2.11 | Up |
| HENMT1 | 0.00000161 | 2.13 | Up |
| MAML2 | 0.00000161 | 1.8 | Up |
| MRC1 | 0.00000166 | 3.02 | Up |
| TNFAIP8 | 0.00000166 | 2.68 | Up |
| DDR2 | 0.00000166 | 2.16 | Up |
| AIM1 | 0.00000168 | 3.06 | Up |
| CSF1R | 0.00000168 | 2.92 | Up |
| OLFML1 | 0.00000168 | 2.87 | Up |
| TWIST1 | 0.00000169 | 2.42 | Up |
| ANTXR2 | 0.0000017 | 2.21 | Up |
| GPR34 | 0.00000172 | 3.3 | Up |
| CYBRD1 | 0.00000175 | 2.94 | Up |
| SIDT2 | 0.00000175 | 1.66 | Up |
| TSPYL4 | 0.00000176 | 1.57 | Up |
| FYN | 0.00000178 | 2.19 | Up |
| LHFP | 0.00000178 | 1.64 | Up |
| ANLN | 0.00000179 | 3.57 | Up |
| EPB41L2 | 0.00000179 | 1.76 | Up |
| CD99 | 0.0000018 | 1.66 | Up |
| PHLDA1 | 0.00000181 | 2.42 | Up |
| PCDH18 | 0.00000182 | 2.97 | Up |
| DAPK1 | 0.00000182 | 2.78 | Up |
| RASA2 | 0.00000183 | 2.01 | Up |
| FAM49B | 0.00000183 | 1.53 | Up |
| HOXB2 | 0.00000184 | 2.16 | Up |
| RGS2 | 0.00000185 | 2.96 | Up |
| SAMHD1 | 0.00000185 | 2.32 | Up |
| SPIN1 | 0.00000185 | 1.93 | Up |
| TMEM35B///ZMYM6 | 0.00000185 | 1.6 | Up |
| TRIM22 | 0.00000192 | 1.8 | Up |
| NMI | 0.00000192 | 1.77 | Up |
| SLC25A43 | 0.00000193 | 2.57 | Up |
| NHSL2 | 0.00000193 | 1.88 | Up |
| HLA-DMB | 0.00000194 | 2.32 | Up |
| CD109 | 0.000002 | 2.68 | Up |
| STMN1 | 0.000002 | 1.72 | Up |
| CSF2RB | 0.00000202 | 3.23 | Up |
| PRTFDC1 | 0.00000202 | 2.92 | Up |
| CNRIP1 | 0.00000204 | 1.88 | Up |
| FCER1G | 0.00000205 | 2.51 | Up |
| SLC16A14 | 0.00000206 | 2.5 | Up |
| NOVA1 | 0.00000206 | 2.35 | Up |
| COL14A1 | 0.00000208 | 4.93 | Up |
| CHSY1 | 0.00000208 | 1.9 | Up |
| CDH11 | 0.00000209 | 3.05 | Up |
| WDR11 | 0.00000209 | 1.62 | Up |
| BNC2 | 0.0000021 | 2.54 | Up |
| PAPSS1 | 0.00000211 | 1.74 | Up |
| TMEM50A | 0.00000218 | 1.52 | Up |
| PIK3CG | 0.00000221 | 2.37 | Up |
| C3orf14 | 0.00000225 | 2.2 | Up |
| PLXDC2 | 0.00000226 | 2.3 | Up |
| GALNT7 | 0.00000227 | 3.53 | Up |
| TGFBR2 | 0.00000227 | 1.83 | Up |
| TMEM243 | 0.0000023 | 1.89 | Up |
| ZNF436 | 0.00000239 | 1.94 | Up |
| PRR11 | 0.00000242 | 2.91 | Up |
| ZFAS1 | 0.00000242 | 1.81 | Up |
| MYCL | 0.00000244 | 2.27 | Up |
| ZNF329 | 0.00000244 | 2.2 | Up |
| BNIP3L | 0.00000244 | 1.67 | Up |
| SLC12A7 | 0.00000245 | 1.85 | Up |
| FST | 0.00000247 | 4.42 | Up |
| ANXA2P2 | 0.00000252 | 1.77 | Up |
| CLEC10A | 0.00000255 | 2.28 | Up |
| SGMS1 | 0.00000255 | 1.62 | Up |
| IRF8 | 0.00000256 | 2.89 | Up |
| GGTA1P | 0.00000263 | 2.16 | Up |
| MMD | 0.0000027 | 2.7 | Up |
| C1S | 0.00000285 | 2.66 | Up |
| EPB41L4A-AS1 | 0.00000287 | 1.78 | Up |
| PHACTR2 | 0.00000291 | 1.59 | Up |
| RAB31 | 0.00000292 | 2.5 | Up |
| TMEM200A | 0.00000296 | 2.66 | Up |
| TCFL5 | 0.00000296 | 1.77 | Up |
| NPM1 | 0.00000298 | 2.44 | Up |
| CENPU | 0.00000306 | 2.68 | Up |
| HYMAI | 0.00000308 | 2.64 | Up |
| ARHGAP36 | 0.00000322 | 5.55 | Up |
| GPC3 | 0.00000331 | 3.11 | Up |
| BCHE | 0.00000331 | 2.63 | Up |
| ZC3HAV1 | 0.00000337 | 1.9 | Up |
| PCDHB14 | 0.0000034 | 2.94 | Up |
| HCLS1 | 0.0000034 | 2.74 | Up |
| TTC8 | 0.00000343 | 1.99 | Up |
| ZWINT | 0.00000344 | 2.8 | Up |
| CREBL2 | 0.00000349 | 1.71 | Up |
| PLEKHA2 | 0.00000354 | 2.03 | Up |
| CXorf38 | 0.0000036 | 1.51 | Up |
| PRPS2 | 0.00000366 | 1.74 | Up |
| LINC01140 | 0.00000369 | 2.5 | Up |
| SOWAHC | 0.00000369 | 1.66 | Up |
| CXCR4 | 0.0000037 | 3.91 | Up |
| CALHM2 | 0.00000376 | 1.61 | Up |
| ZFP82 | 0.00000378 | 1.79 | Up |
| ANXA1 | 0.00000383 | 1.99 | Up |
| DOCK10 | 0.00000386 | 2.21 | Up |
| NOTCH2NL | 0.00000388 | 2.38 | Up |
| STAG3L2///STAG3L3///TRIM73 | 0.00000393 | 2.3 | Up |
| TGFBR3 | 0.00000403 | 1.73 | Up |
| MFSD1 | 0.00000404 | 1.57 | Up |
| PLPPR4 | 0.0000041 | 3.36 | Up |
| SYTL2 | 0.00000412 | 3.16 | Up |
| MIR4680///PDCD4 | 0.00000416 | 1.56 | Up |
| PYCARD | 0.00000421 | 2.87 | Up |
| CFH | 0.00000423 | 3.19 | Up |
| UGCG | 0.00000428 | 2.45 | Up |
| KPNA2 | 0.00000429 | 2.04 | Up |
| BID | 0.00000437 | 2.14 | Up |
| TWSG1 | 0.00000451 | 1.56 | Up |
| MEIS2 | 0.00000454 | 2.66 | Up |
| MVP | 0.00000457 | 1.56 | Up |
| MARVELD1 | 0.00000458 | 2.34 | Up |
| CECR1 | 0.00000461 | 3.44 | Up |
| PARVA | 0.00000461 | 1.67 | Up |
| TNFSF12-TNFSF13///TNFSF13 | 0.00000462 | 1.76 | Up |
| GLIPR2 | 0.00000469 | 2.08 | Up |
| SRGAP2C///SRGAP2B///SRGAP2 | 0.00000469 | 1.58 | Up |
| CTSC | 0.00000475 | 1.8 | Up |
| LINC01279 | 0.00000481 | 4.72 | Up |
| SMAD1 | 0.00000481 | 1.97 | Up |
| CD14 | 0.00000485 | 3.46 | Up |
| B3GALNT1 | 0.00000488 | 1.69 | Up |
| MSR1 | 0.00000492 | 3.03 | Up |
| MAN1C1 | 0.00000493 | 2 | Up |
| EDNRB | 0.00000503 | 1.65 | Up |
| TMEM173 | 0.00000514 | 2.28 | Up |
| ISLR | 0.00000514 | 2.27 | Up |
| NRN1 | 0.00000523 | 1.58 | Up |
| 1-Mar | 0.00000525 | 2.45 | Up |
| RAI14 | 0.00000542 | 2.3 | Up |
| CPNE8 | 0.00000545 | 1.58 | Up |
| MKNK1 | 0.0000055 | 2.03 | Up |
| SERPING1 | 0.00000557 | 1.99 | Up |
| RUNX1-IT1 | 0.00000564 | 3.7 | Up |
| BTN3A3 | 0.00000566 | 1.61 | Up |
| RALGPS2 | 0.00000569 | 2.45 | Up |
| ECM2 | 0.00000582 | 2.72 | Up |
| LAIR1 | 0.00000585 | 3.13 | Up |
| PIGB | 0.0000059 | 1.51 | Up |
| C17orf58 | 0.00000601 | 1.51 | Up |
| MOB1B | 0.0000061 | 1.62 | Up |
| GJA1 | 0.00000626 | 2.3 | Up |
| IFI44 | 0.00000636 | 2.88 | Up |
| CLEC11A | 0.00000642 | 3.13 | Up |
| IFI44L | 0.00000643 | 3.09 | Up |
| CLIC2 | 0.00000643 | 1.55 | Up |
| ANXA2 | 0.00000662 | 1.71 | Up |
| ARPP21 | 0.00000664 | 3.86 | Up |
| MDM1 | 0.0000068 | 1.92 | Up |
| ATP8B4 | 0.00000692 | 2.88 | Up |
| PRMT2 | 0.00000692 | 1.55 | Up |
| LRP11 | 0.00000696 | 2.27 | Up |
| RPL22L1 | 0.00000702 | 2.08 | Up |
| VAMP8 | 0.00000704 | 1.89 | Up |
| PTTG1 | 0.00000709 | 2.83 | Up |
| CCR1 | 0.00000719 | 3.03 | Up |
| C1orf105 | 0.00000722 | 4.2 | Up |
| RNF135 | 0.00000726 | 1.66 | Up |
| TSPAN4 | 0.00000738 | 2.5 | Up |
| IL10RA | 0.0000074 | 3.31 | Up |
| CDO1 | 0.00000743 | 1.85 | Up |
| GIMAP7 | 0.00000759 | 2.04 | Up |
| TCTEX1D2 | 0.00000761 | 1.6 | Up |
| NBPF26///NBPF10///NBPF9///NBPF14 | 0.00000766 | 1.54 | Up |
| DDAH2 | 0.00000807 | 2.11 | Up |
| CTSO | 0.00000813 | 1.66 | Up |
| KCNMB4 | 0.00000822 | 2.06 | Up |
| NARR///RAB34 | 0.00000826 | 2.01 | Up |
| ZNF711 | 0.00000826 | 1.86 | Up |
| SUCO | 0.0000084 | 2.03 | Up |
| TPST1 | 0.00000863 | 2.44 | Up |
| TIMP1 | 0.00000869 | 3.01 | Up |
| S100A10 | 0.00000877 | 1.94 | Up |
| IFI30///PIK3R2 | 0.00000879 | 2.87 | Up |
| SPX | 0.00000881 | 1.73 | Up |
| PGM2 | 0.00000889 | 1.92 | Up |
| RARRES2 | 0.00000893 | 2.57 | Up |
| NABP1 | 0.00000908 | 2.58 | Up |
| GADD45A | 0.0000092 | 3.45 | Up |
| KIAA0101 | 0.00000924 | 3.69 | Up |
| ANKRD10 | 0.00000928 | 1.54 | Up |
| PDGFRL | 0.00000934 | 2.49 | Up |
| PRDM1 | 0.00000934 | 1.7 | Up |
| NCKAP1L | 0.0000094 | 2.34 | Up |
| IQGAP2 | 0.00000943 | 3.08 | Up |
| FAP | 0.00000943 | 2.57 | Up |
| CDC25B | 0.00000948 | 2.51 | Up |
| LGALS3 | 0.00000957 | 1.64 | Up |
| SLC7A7 | 0.00000981 | 3.15 | Up |
| TTLL11 | 0.00000997 | 1.72 | Up |
| SAMSN1 | 0.0000101 | 3.4 | Up |
| PRRX1 | 0.0000101 | 2.32 | Up |
| UGDH | 0.0000101 | 1.84 | Up |
| IFIT1 | 0.0000101 | 1.63 | Up |
| AJUBA | 0.0000103 | 3.08 | Up |
| LIPA | 0.0000104 | 2.27 | Up |
| TASP1 | 0.0000104 | 1.54 | Up |
| TMEM119 | 0.0000105 | 2.8 | Up |
| RBBP8 | 0.0000107 | 1.92 | Up |
| SCN7A | 0.0000107 | 1.51 | Up |
| SLC16A2 | 0.0000109 | 1.73 | Up |
| PLSCR1 | 0.000011 | 1.92 | Up |
| LINC00936 | 0.000011 | 1.61 | Up |
| TPPP3 | 0.0000111 | 2.41 | Up |
| HLA-DMA | 0.0000111 | 2.4 | Up |
| FAM107B | 0.0000111 | 1.75 | Up |
| SP110 | 0.0000112 | 1.77 | Up |
| CD53 | 0.0000113 | 2.51 | Up |
| KDELR3 | 0.0000114 | 2.78 | Up |
| ZNF608 | 0.0000115 | 1.87 | Up |
| SEC23B | 0.0000117 | 1.89 | Up |
| SRGN | 0.0000118 | 1.58 | Up |
| FBN1 | 0.0000119 | 2.39 | Up |
| CSTA | 0.0000123 | 2.72 | Up |
| EBF1 | 0.0000124 | 2.51 | Up |
| NDN | 0.0000124 | 2.2 | Up |
| OLFML2A | 0.0000124 | 2.05 | Up |
| TUBB | 0.0000124 | 1.59 | Up |
| NXT2 | 0.0000124 | 1.54 | Up |
| EVI2B | 0.0000125 | 3.04 | Up |
| CAPG | 0.0000125 | 2.9 | Up |
| RBM3 | 0.0000125 | 1.81 | Up |
| MGC24103 | 0.0000128 | 2.77 | Up |
| MIR99AHG | 0.0000128 | 1.74 | Up |
| RNASE4 | 0.0000134 | 2.43 | Up |
| RAPH1 | 0.0000134 | 1.93 | Up |
| C1orf54 | 0.0000139 | 1.58 | Up |
| MYD88 | 0.0000141 | 2.28 | Up |
| TRPC1 | 0.0000141 | 2.26 | Up |
| SOAT1 | 0.0000141 | 2.03 | Up |
| TRA2A | 0.0000141 | 1.62 | Up |
| CLEC4A | 0.0000142 | 2.49 | Up |
| MDK | 0.0000145 | 1.83 | Up |
| HOTAIRM1 | 0.0000146 | 1.98 | Up |
| ZNF268 | 0.0000146 | 1.91 | Up |
| NT5E | 0.0000147 | 2.88 | Up |
| IGFBP3 | 0.0000148 | 3.74 | Up |
| HMCN1 | 0.0000151 | 1.75 | Up |
| RND3 | 0.0000152 | 2.01 | Up |
| SNX10 | 0.0000152 | 1.69 | Up |
| CPVL | 0.0000154 | 1.99 | Up |
| MPZL1 | 0.0000154 | 1.84 | Up |
| EVI2A | 0.0000155 | 3.18 | Up |
| RNF217 | 0.0000158 | 1.52 | Up |
| ZNF83 | 0.0000162 | 2.06 | Up |
| ZNF700 | 0.0000162 | 1.51 | Up |
| ITGBL1 | 0.0000164 | 2.8 | Up |
| MID1 | 0.0000165 | 2.55 | Up |
| FAM102B | 0.0000168 | 2.63 | Up |
| DACT1 | 0.000017 | 3.65 | Up |
| LAMP2 | 0.000017 | 1.64 | Up |
| C10orf10 | 0.0000171 | 2.37 | Up |
| CLMP | 0.0000173 | 2.47 | Up |
| PCDHB9///PCDHB10 | 0.0000176 | 2.74 | Up |
| SMAP2 | 0.0000176 | 1.53 | Up |
| PRR5 | 0.0000181 | 1.56 | Up |
| WRN | 0.0000181 | 1.52 | Up |
| MIR21///VMP1 | 0.0000182 | 3.8 | Up |
| DMRT2 | 0.0000182 | 2.23 | Up |
| GLYR1///SEPT6 | 0.0000186 | 2.15 | Up |
| P2RX4 | 0.0000188 | 1.72 | Up |
| C1QA | 0.0000191 | 2.67 | Up |
| PLTP | 0.0000191 | 2.4 | Up |
| KLHL2 | 0.0000191 | 1.92 | Up |
| C17orf62 | 0.0000191 | 1.85 | Up |
| ITIH5 | 0.0000194 | 1.58 | Up |
| DEPDC7 | 0.0000195 | 2.19 | Up |
| PLSCR4 | 0.0000196 | 1.91 | Up |
| VAV3 | 0.0000196 | 1.51 | Up |
| CKLF-CMTM1///CKLF | 0.0000199 | 2.21 | Up |
| SULF2 | 0.0000199 | 1.82 | Up |
| EPSTI1 | 0.00002 | 2.26 | Up |
| PRKACB | 0.0000201 | 1.66 | Up |
| GIMAP8 | 0.0000202 | 1.57 | Up |
| S100A16 | 0.0000204 | 1.77 | Up |
| CMKLR1 | 0.0000204 | 1.61 | Up |
| PALM2 | 0.0000209 | 2.3 | Up |
| EMB | 0.0000212 | 2.61 | Up |
| ACKR1 | 0.0000219 | 2.67 | Up |
| LIMA1 | 0.0000219 | 1.55 | Up |
| FSTL1 | 0.0000222 | 1.88 | Up |
| FAM117A | 0.0000222 | 1.84 | Up |
| LPXN | 0.0000223 | 1.89 | Up |
| VSIG4 | 0.0000225 | 2.75 | Up |
| NES | 0.0000225 | 2.05 | Up |
| TP53INP1 | 0.0000228 | 1.52 | Up |
| TTC9 | 0.0000234 | 1.87 | Up |
| IL32 | 0.0000236 | 3.18 | Up |
| P3H1 | 0.0000245 | 1.79 | Up |
| PDGFD | 0.0000247 | 3.04 | Up |
| APOC1 | 0.0000248 | 3.06 | Up |
| LINC01116 | 0.000025 | 1.77 | Up |
| S100A11 | 0.0000252 | 1.59 | Up |
| HHEX | 0.0000254 | 1.54 | Up |
| SNHG24 | 0.0000257 | 3.28 | Up |
| STMN2 | 0.0000266 | 3.81 | Up |
| CXCL14 | 0.0000268 | 2.7 | Up |
| BICC1 | 0.0000273 | 1.75 | Up |
| TMEM14A | 0.0000276 | 1.52 | Up |
| CD1C | 0.0000277 | 2.48 | Up |
| RAB27A | 0.0000279 | 1.67 | Up |
| LYZ | 0.0000282 | 3.64 | Up |
| NPL | 0.0000287 | 2.22 | Up |
| VIM | 0.0000289 | 1.67 | Up |
| MYL4 | 0.0000291 | 2.13 | Up |
| CPA3 | 0.0000293 | 3.11 | Up |
| FAM13A-AS1 | 0.0000293 | 2.02 | Up |
| LAYN | 0.0000304 | 2.25 | Up |
| LINC00597 | 0.0000308 | 2.51 | Up |
| S100A4 | 0.0000311 | 2 | Up |
| SOCS2 | 0.0000316 | 2.2 | Up |
| MAFB | 0.0000321 | 1.51 | Up |
| ADCY3 | 0.0000327 | 1.8 | Up |
| F2R | 0.0000327 | 1.78 | Up |
| MGP | 0.0000328 | 2.34 | Up |
| CPXM1 | 0.0000329 | 2.94 | Up |
| DAPK1-IT1 | 0.0000332 | 1.79 | Up |
| MAL | 0.0000341 | 2.26 | Up |
| LRRN4CL | 0.0000341 | 1.73 | Up |
| ARL6IP1 | 0.0000341 | 1.63 | Up |
| SHE | 0.0000341 | 1.58 | Up |
| DOCK11 | 0.0000343 | 2.65 | Up |
| SDC2 | 0.0000354 | 2.1 | Up |
| TLR5 | 0.0000354 | 1.95 | Up |
| AGTR1 | 0.000036 | 2.11 | Up |
| HGF | 0.0000364 | 2.4 | Up |
| DTL | 0.0000369 | 2.21 | Up |
| HRH1 | 0.0000377 | 2.08 | Up |
| ZNF559-ZNF177///ZNF177 | 0.0000381 | 2.15 | Up |
| HS6ST2 | 0.0000382 | 2.9 | Up |
| PRC1 | 0.0000383 | 1.9 | Up |
| RUNX1T1 | 0.0000385 | 1.56 | Up |
| GAS2L3 | 0.0000391 | 1.94 | Up |
| SLCO2B1 | 0.0000393 | 2.09 | Up |
| TMEM176A | 0.0000394 | 2.08 | Up |
| GPC4 | 0.0000399 | 1.68 | Up |
| STEAP4 | 0.0000403 | 1.66 | Up |
| ITM2A | 0.0000414 | 1.91 | Up |
| RAB11FIP3 | 0.0000415 | 1.59 | Up |
| MTMR9 | 0.0000419 | 2.27 | Up |
| RAMP3 | 0.0000419 | 1.75 | Up |
| PLAU | 0.0000425 | 2.71 | Up |
| ZNF559 | 0.0000425 | 1.81 | Up |
| C3AR1 | 0.0000426 | 2.39 | Up |
| ZNF431 | 0.0000426 | 1.89 | Up |
| HERC5 | 0.0000427 | 1.84 | Up |
| EHD4 | 0.0000432 | 1.55 | Up |
| LACC1 | 0.0000434 | 2.55 | Up |
| FCGR2B | 0.0000436 | 2.98 | Up |
| MAP1LC3C | 0.0000438 | 2.22 | Up |
| GBP1 | 0.0000447 | 1.87 | Up |
| PLP2 | 0.0000452 | 1.57 | Up |
| PCDH17 | 0.0000453 | 1.83 | Up |
| ZNF286B///ZNF286A | 0.0000462 | 1.84 | Up |
| GEMIN2 | 0.0000465 | 1.58 | Up |
| IFFO1 | 0.0000471 | 1.74 | Up |
| ST8SIA4 | 0.0000472 | 1.91 | Up |
| KYNU | 0.0000474 | 2.27 | Up |
| SCUBE2 | 0.0000478 | 1.88 | Up |
| GORAB | 0.0000478 | 1.77 | Up |
| SLFN11 | 0.0000493 | 1.82 | Up |
| GALNT10 | 0.0000499 | 1.6 | Up |
| RAC2 | 0.0000506 | 1.83 | Up |
| TLR7 | 0.0000507 | 2.24 | Up |
| FPR3 | 0.0000508 | 3.13 | Up |
| TUBB2B | 0.0000508 | 1.54 | Up |
| ERRFI1 | 0.000052 | 2.44 | Up |
| ARPC1B | 0.0000521 | 1.76 | Up |
| RCBTB2 | 0.0000521 | 1.66 | Up |
| TRAF5 | 0.0000523 | 2.22 | Up |
| ROR1 | 0.0000529 | 1.8 | Up |
| WDFY2 | 0.0000537 | 2.04 | Up |
| PSAT1 | 0.000054 | 1.73 | Up |
| C16orf54 | 0.0000542 | 1.93 | Up |
| PPIB | 0.0000552 | 1.61 | Up |
| MME | 0.0000552 | 1.54 | Up |
| CCR5 | 0.0000554 | 1.85 | Up |
| FLVCR1 | 0.0000571 | 1.68 | Up |
| LY86 | 0.0000574 | 2.09 | Up |
| WASF1 | 0.0000574 | 1.66 | Up |
| FBXL13 | 0.0000584 | 2.54 | Up |
| ASS1 | 0.0000584 | 2.19 | Up |
| RIMKLB | 0.0000591 | 1.67 | Up |
| PXDN | 0.0000594 | 1.72 | Up |
| F13A1 | 0.0000601 | 3.21 | Up |
| ADAMTS9 | 0.0000602 | 2.13 | Up |
| THSD7A | 0.0000603 | 1.96 | Up |
| ACSL4 | 0.0000605 | 1.82 | Up |
| S100A2 | 0.0000606 | 3.99 | Up |
| SFRP4 | 0.0000616 | 3.2 | Up |
| NCF2 | 0.0000616 | 2.43 | Up |
| BGN | 0.0000619 | 2.64 | Up |
| SIGLEC1 | 0.0000619 | 2.24 | Up |
| FN1 | 0.0000626 | 1.87 | Up |
| APOE | 0.0000627 | 2.38 | Up |
| PVALB | 0.000063 | 5.08 | Up |
| CLSTN2 | 0.000063 | 2.59 | Up |
| KLF5 | 0.0000631 | 2.32 | Up |
| TRIL | 0.0000633 | 1.55 | Up |
| ARG2 | 0.0000638 | 3.81 | Up |
| DPP4 | 0.000064 | 2.2 | Up |
| SNORD114-3 | 0.0000641 | 3.01 | Up |
| SYK | 0.0000657 | 1.73 | Up |
| ANKRD29 | 0.0000668 | 1.57 | Up |
| CD52 | 0.0000669 | 1.88 | Up |
| XRCC4 | 0.0000673 | 2.82 | Up |
| DUSP6 | 0.0000698 | 1.56 | Up |
| TMTC2 | 0.00007 | 1.68 | Up |
| RGS10 | 0.0000702 | 2.02 | Up |
| MINOS1-NBL1///NBL1 | 0.0000706 | 1.81 | Up |
| SLC43A3 | 0.0000707 | 1.58 | Up |
| SRGAP2 | 0.0000713 | 1.76 | Up |
| MIR7110///PDIA5 | 0.0000721 | 1.65 | Up |
| SAMD9 | 0.0000726 | 1.99 | Up |
| SERTAD4 | 0.0000754 | 1.82 | Up |
| HMGCLL1 | 0.0000767 | 2.16 | Up |
| PLAUR | 0.0000773 | 2.38 | Up |
| PSMB9 | 0.0000779 | 1.6 | Up |
| PTGFR | 0.000079 | 2.42 | Up |
| RBP7 | 0.0000806 | 2.33 | Up |
| GLIPR1 | 0.0000812 | 2.38 | Up |
| ALCAM | 0.0000837 | 2.12 | Up |
| CAPRIN2 | 0.0000841 | 1.58 | Up |
| LSP1 | 0.0000847 | 1.59 | Up |
| EBF2 | 0.0000848 | 1.69 | Up |
| SERPINB1 | 0.0000848 | 1.51 | Up |
| DDX60L | 0.000086 | 1.85 | Up |
| HCK | 0.0000879 | 1.8 | Up |
| FNBP1 | 0.0000886 | 1.53 | Up |
| GFRA1 | 0.0000902 | 1.56 | Up |
| PROM1 | 0.0000903 | 1.91 | Up |
| C1QTNF2 | 0.0000912 | 1.89 | Up |
| PRICKLE2 | 0.0000912 | 1.78 | Up |
| SERPINI1 | 0.0000916 | 1.64 | Up |
| IRF7 | 0.000094 | 1.84 | Up |
| ID2B///ID2 | 0.0000959 | 2.17 | Up |
| NTN4 | 0.0000981 | 1.74 | Up |
| ADRA2A | 0.0000992 | 2.82 | Up |
| SOX8 | 0.000101 | 2.82 | Up |
| TET1 | 0.000101 | 1.56 | Up |
| CADM2 | 0.000103 | 2.37 | Up |
| NCF4 | 0.000103 | 1.81 | Up |
| HACD2 | 0.000106 | 1.82 | Up |
| LCP1 | 0.00011 | 2.16 | Up |
| JAZF1 | 0.00011 | 1.77 | Up |
| OSMR | 0.000112 | 2.38 | Up |
| MSC-AS1 | 0.000112 | 1.74 | Up |
| NEGR1 | 0.000112 | 1.72 | Up |
| PRKAR2B | 0.000113 | 2.9 | Up |
| CPE | 0.000118 | 1.52 | Up |
| ATP10D | 0.000119 | 1.81 | Up |
| P3H4 | 0.000121 | 1.52 | Up |
| SULF1 | 0.000123 | 2.68 | Up |
| ELL3 | 0.000124 | 2.04 | Up |
| KDELC1 | 0.000124 | 1.8 | Up |
| GGH | 0.000126 | 1.62 | Up |
| CMPK2 | 0.000129 | 1.72 | Up |
| C1QB | 0.000131 | 2.79 | Up |
| CEP192 | 0.000131 | 1.59 | Up |
| ANKRD36B | 0.000132 | 1.59 | Up |
| ELN | 0.000137 | 2.37 | Up |
| GMFG | 0.000138 | 1.64 | Up |
| NAP1L3 | 0.000139 | 2.11 | Up |
| RTN1 | 0.00014 | 2.17 | Up |
| ACKR3 | 0.000146 | 2.23 | Up |
| CARD6 | 0.000153 | 1.8 | Up |
| ANG | 0.000153 | 1.57 | Up |
| KCNT2 | 0.000154 | 1.7 | Up |
| IL17B | 0.000155 | 3.48 | Up |
| LINC00094 | 0.000155 | 1.95 | Up |
| CYSLTR1 | 0.000164 | 2.25 | Up |
| SNORA11E///SNORA11D///MAGED4///MAGED4B | 0.00017 | 1.93 | Up |
| ZNF117 | 0.00017 | 1.74 | Up |
| WDR19 | 0.00017 | 1.66 | Up |
| ID2 | 0.000175 | 1.56 | Up |
| METTL21B | 0.000177 | 1.67 | Up |
| MAGED2 | 0.000179 | 1.58 | Up |
| CEP126 | 0.000181 | 1.71 | Up |
| PTPRF | 0.000189 | 2.1 | Up |
| TGFBI | 0.000189 | 1.62 | Up |
| GPC6 | 0.00019 | 2.93 | Up |
| FABP4 | 0.000195 | 1.8 | Up |
| TBC1D8B | 0.000197 | 1.58 | Up |
| SLC16A4 | 0.000198 | 1.77 | Up |
| PGBD5 | 0.0002 | 1.9 | Up |
| RBP1 | 0.000202 | 1.75 | Up |
| COL21A1 | 0.000205 | 2.51 | Up |
| CD24 | 0.00022 | 2.64 | Up |
| CDC42EP5 | 0.00022 | 1.54 | Up |
| MAMDC2 | 0.000228 | 1.7 | Up |
| CDKN1A | 0.00023 | 2.65 | Up |
| PPM1M | 0.000241 | 1.52 | Up |
| KIT | 0.000243 | 1.61 | Up |
| TRIM38 | 0.000247 | 1.96 | Up |
| GZMA | 0.000248 | 2.06 | Up |
| PLEK | 0.00025 | 1.57 | Up |
| NBPF20 | 0.000258 | 1.58 | Up |
| SERPINA3 | 0.000261 | 3.21 | Up |
| DUBR | 0.000266 | 1.68 | Up |
| MYF5 | 0.000269 | 2.02 | Up |
| BTN3A2 | 0.000273 | 1.88 | Up |
| INS-IGF2///IGF2 | 0.000273 | 1.83 | Up |
| MAST3 | 0.000281 | 1.84 | Up |
| NBL1 | 0.000281 | 1.81 | Up |
| FAM20A | 0.000285 | 1.55 | Up |
| ST8SIA1 | 0.000291 | 1.67 | Up |
| FGF2 | 0.000292 | 1.93 | Up |
| MAMLD1 | 0.000294 | 1.54 | Up |
| SLA | 0.000301 | 1.94 | Up |
| SLC22A4 | 0.000302 | 1.63 | Up |
| FAM109B | 0.000302 | 1.55 | Up |
| TRO | 0.000307 | 2.11 | Up |
| MX2 | 0.000308 | 2.13 | Up |
| SLC27A3 | 0.000312 | 1.68 | Up |
| DBN1 | 0.000313 | 1.57 | Up |
| SNX7 | 0.00032 | 2.27 | Up |
| ADAMTS5 | 0.000327 | 2.06 | Up |
| FXYD5 | 0.000327 | 1.95 | Up |
| TUFT1 | 0.000331 | 1.54 | Up |
| CD99P1 | 0.000332 | 1.85 | Up |
| FBLN1 | 0.000342 | 2.28 | Up |
| C1QC | 0.000348 | 2.57 | Up |
| ZNF556 | 0.000368 | 2.07 | Up |
| KIAA0226L | 0.000376 | 1.99 | Up |
| MFAP4 | 0.000376 | 1.93 | Up |
| STK39 | 0.000376 | 1.66 | Up |
| MYO1F | 0.000377 | 1.7 | Up |
| AOC3 | 0.000379 | 1.62 | Up |
| CCL5 | 0.00038 | 2.13 | Up |
| NAIP | 0.000401 | 1.69 | Up |
| GAS7 | 0.000409 | 1.54 | Up |
| LILRB2 | 0.000416 | 2.39 | Up |
| EZH2 | 0.000427 | 1.68 | Up |
| LRRN3 | 0.000429 | 2.79 | Up |
| TCEAL7 | 0.000441 | 2.05 | Up |
| VMP1 | 0.000446 | 3.09 | Up |
| THEMIS2 | 0.00045 | 1.76 | Up |
| TLR1 | 0.000451 | 2.05 | Up |
| SERPINH1 | 0.000456 | 1.73 | Up |
| SLC39A10 | 0.000462 | 1.85 | Up |
| ARL4A | 0.000463 | 1.9 | Up |
| PROCR | 0.000475 | 1.73 | Up |
| XG | 0.000509 | 2.55 | Up |
| SH3PXD2B | 0.000528 | 1.74 | Up |
| EXOSC7///CLEC3B | 0.000544 | 1.63 | Up |
| YPEL1 | 0.00055 | 1.57 | Up |
| IFIT3 | 0.000551 | 1.85 | Up |
| CCL2 | 0.000559 | 3.15 | Up |
| GNA14 | 0.000578 | 1.67 | Up |
| SLC38A9 | 0.000583 | 1.62 | Up |
| EML6 | 0.000584 | 1.51 | Up |
| SHD | 0.00059 | 2.38 | Up |
| RSAD2 | 0.000592 | 1.65 | Up |
| INSIG1 | 0.000592 | 1.53 | Up |
| ADAMTSL3 | 0.000618 | 1.52 | Up |
| GEM | 0.000626 | 1.62 | Up |
| LST1 | 0.000633 | 1.62 | Up |
| EN1 | 0.000639 | 1.7 | Up |
| GBP3 | 0.000642 | 2.11 | Up |
| FREM1 | 0.000654 | 1.54 | Up |
| MCTP1 | 0.00073 | 2.87 | Up |
| UHRF1 | 0.000743 | 1.81 | Up |
| CCR2 | 0.00077 | 1.69 | Up |
| DKK2 | 0.000771 | 2.52 | Up |
| CPXM2 | 0.000791 | 2.01 | Up |
| LINC00844 | 0.000824 | 3.56 | Up |
| IFIT2 | 0.000849 | 1.6 | Up |
| CCDC169-SOHLH2///SOHLH2 | 0.000856 | 1.98 | Up |
| EBPL | 0.000878 | 1.62 | Up |
| CCL18 | 0.000927 | 2.48 | Up |
| BEX2 | 0.000941 | 1.75 | Up |
| DMBT1 | 0.000971 | 2.32 | Up |
| LINC00342 | 0.000999 | 1.96 | Up |
| IRX5 | 5.18E-14 | -4.34 | Down |
| CFAP46 | 1.29E-10 | -3.88 | Down |
| SLC1A4 | 2.11E-10 | -2.96 | Down |
| TYRP1 | 3.15E-10 | -5.72 | Down |
| FAM179A | 1.05E-09 | -4.05 | Down |
| MLXIP | 3.17E-09 | -2.53 | Down |
| RNF126 | 8.83E-09 | -1.64 | Down |
| CTU2 | 9.61E-09 | -1.66 | Down |
| MAN2A2 | 1.52E-08 | -1.72 | Down |
| SSH1 | 2.07E-08 | -2.1 | Down |
| SAMD4A | 3.02E-08 | -2.88 | Down |
| IQSEC2 | 4.02E-08 | -2.07 | Down |
| LGI1 | 4.79E-08 | -4.5 | Down |
| ETNPPL | 0.000000049 | -4.54 | Down |
| PWWP2B | 5.08E-08 | -1.93 | Down |
| MPPED2 | 5.41E-08 | -3.9 | Down |
| MYH14 | 0.000000066 | -2.46 | Down |
| FLII | 0.000000066 | -1.84 | Down |
| RAD23A | 6.73E-08 | -1.86 | Down |
| DNAJC12 | 6.84E-08 | -3.6 | Down |
| PAQR9 | 8.97E-08 | -4.7 | Down |
| EXTL3 | 9.17E-08 | -1.71 | Down |
| ABTB2 | 0.000000119 | -2.93 | Down |
| SCN1B | 0.00000014 | -2.2 | Down |
| MBNL1-AS1 | 0.000000173 | -1.6 | Down |
| MIR29C///MIR29B2 | 0.000000178 | -2.32 | Down |
| MBNL1 | 0.000000181 | -2 | Down |
| TTC39A | 0.000000187 | -1.64 | Down |
| GATSL2 | 0.000000199 | -1.71 | Down |
| SLC26A9 | 0.000000201 | -4.47 | Down |
| C1orf127 | 0.000000207 | -3.02 | Down |
| ATP1A2 | 0.000000207 | -1.62 | Down |
| KIF1C | 0.000000226 | -1.83 | Down |
| MSRB2 | 0.000000226 | -1.72 | Down |
| OR7E12P | 0.000000236 | -1.98 | Down |
| RNF123 | 0.000000239 | -1.66 | Down |
| TMEM110 | 0.00000024 | -1.81 | Down |
| PPP3CC | 0.000000262 | -1.52 | Down |
| RXRA | 0.000000271 | -1.77 | Down |
| SCGB1D2 | 0.000000281 | -4.18 | Down |
| UBE2D4 | 0.000000295 | -1.65 | Down |
| ESRRA | 0.000000317 | -1.59 | Down |
| CYP4B1 | 0.000000323 | -3.25 | Down |
| ATG4D | 0.000000334 | -1.8 | Down |
| SLC25A23 | 0.000000339 | -1.63 | Down |
| C6orf136 | 0.000000339 | -1.59 | Down |
| SAMD14 | 0.000000341 | -1.52 | Down |
| SBF1 | 0.000000346 | -1.8 | Down |
| SNTA1 | 0.000000351 | -2.13 | Down |
| LGR5 | 0.000000387 | -3.61 | Down |
| BCL7B | 0.000000396 | -1.62 | Down |
| PDLIM2 | 0.000000412 | -2.02 | Down |
| ST3GAL1 | 0.000000435 | -2.14 | Down |
| ABCB4 | 0.000000436 | -2.85 | Down |
| ACACB | 0.000000466 | -1.81 | Down |
| ZBTB47 | 0.000000467 | -1.7 | Down |
| SFXN4 | 0.000000467 | -1.68 | Down |
| SH2B2 | 0.000000485 | -1.97 | Down |
| ABCB4///ABCB1 | 0.000000517 | -2.35 | Down |
| PAM16 | 0.000000518 | -1.58 | Down |
| MAOB | 0.000000535 | -2.01 | Down |
| INTS1 | 0.000000539 | -1.64 | Down |
| SMIM1 | 0.00000055 | -1.91 | Down |
| NDUFAF4 | 0.000000578 | -1.92 | Down |
| TLE2 | 0.000000599 | -2.06 | Down |
| GMPR | 0.000000599 | -1.63 | Down |
| AMIGO1 | 0.000000625 | -2.04 | Down |
| VTA1 | 0.000000631 | -1.52 | Down |
| RNF157 | 0.000000638 | -2.41 | Down |
| NFATC1 | 0.00000064 | -2.06 | Down |
| SLC6A8 | 0.000000666 | -1.81 | Down |
| TMEM110-MUSTN1///TMEM110 | 0.000000725 | -2.48 | Down |
| ACOT11 | 0.000000741 | -2.29 | Down |
| PERM1 | 0.000000838 | -2.9 | Down |
| GPR157 | 0.000000839 | -2.71 | Down |
| RGS3 | 0.000000875 | -2.01 | Down |
| FBXW5 | 0.00000091 | -1.52 | Down |
| MYLK4 | 0.000000925 | -4.16 | Down |
| PGPEP1 | 0.000000947 | -1.91 | Down |
| MAVS | 0.000000948 | -1.87 | Down |
| NOS1 | 0.00000102 | -3.61 | Down |
| ZFP91 | 0.00000102 | -1.8 | Down |
| PTP4A1 | 0.00000103 | -1.55 | Down |
| TWF2 | 0.00000107 | -1.69 | Down |
| PINK1 | 0.00000108 | -1.53 | Down |
| RABL6 | 0.00000113 | -2.05 | Down |
| TBC1D1 | 0.00000116 | -1.82 | Down |
| NR2F6 | 0.00000117 | -2.22 | Down |
| JMJD6 | 0.00000122 | -1.85 | Down |
| NRG4 | 0.00000126 | -2.76 | Down |
| FBXO40 | 0.00000144 | -1.9 | Down |
| PARVB | 0.00000155 | -2.13 | Down |
| ZNF628 | 0.00000157 | -1.75 | Down |
| SLC25A12 | 0.00000157 | -1.56 | Down |
| MN1 | 0.00000158 | -2.35 | Down |
| FHOD1 | 0.00000159 | -1.79 | Down |
| OR7E47P | 0.00000162 | -4.12 | Down |
| NKX3-1 | 0.00000168 | -1.77 | Down |
| DNAJB5 | 0.00000174 | -2.63 | Down |
| FAM222B | 0.00000179 | -1.64 | Down |
| TPM2 | 0.00000181 | -2.61 | Down |
| GAS2 | 0.00000182 | -3.27 | Down |
| FRMD3 | 0.00000182 | -2.28 | Down |
| MAP1S | 0.00000183 | -1.59 | Down |
| SLC38A3 | 0.00000184 | -2.8 | Down |
| CHRNA10 | 0.00000187 | -1.97 | Down |
| ZBTB16 | 0.00000187 | -1.95 | Down |
| KCNJ11 | 0.00000192 | -2.64 | Down |
| DHCR24 | 0.00000194 | -3.53 | Down |
| HSPB7 | 0.00000194 | -1.7 | Down |
| ZNF579 | 0.00000201 | -1.9 | Down |
| PCIF1 | 0.00000205 | -1.92 | Down |
| PSTPIP2 | 0.00000222 | -2.27 | Down |
| PLEC | 0.00000224 | -3.03 | Down |
| PNPLA6 | 0.00000226 | -1.64 | Down |
| PITX1 | 0.00000233 | -2.7 | Down |
| CA2 | 0.00000246 | -2.49 | Down |
| AKR1B10 | 0.00000254 | -2.58 | Down |
| HPN | 0.00000255 | -2.2 | Down |
| CHMP4B | 0.00000255 | -1.86 | Down |
| MAPT | 0.00000256 | -2.2 | Down |
| SRF | 0.00000262 | -2.41 | Down |
| LONRF2 | 0.00000277 | -3.03 | Down |
| DGKZ | 0.00000279 | -2.62 | Down |
| EPM2A | 0.00000287 | -1.51 | Down |
| SOX9-AS1 | 0.00000288 | -2.72 | Down |
| COLQ | 0.0000031 | -2.3 | Down |
| POLR1E | 0.0000031 | -1.78 | Down |
| PGP | 0.00000315 | -1.73 | Down |
| POLRMT | 0.0000032 | -1.6 | Down |
| FKBP5 | 0.0000033 | -3.46 | Down |
| DCUN1D2 | 0.00000344 | -1.64 | Down |
| ASB2 | 0.00000346 | -2.4 | Down |
| AGPAT3 | 0.00000348 | -1.73 | Down |
| RNF34 | 0.00000357 | -1.53 | Down |
| TBX1 | 0.0000036 | -3.99 | Down |
| SEMA6D | 0.0000036 | -1.81 | Down |
| NACC1 | 0.00000368 | -1.55 | Down |
| EPN1 | 0.00000379 | -1.75 | Down |
| FNDC5 | 0.00000386 | -2.18 | Down |
| TBC1D8 | 0.00000387 | -1.79 | Down |
| PNPLA2 | 0.0000041 | -1.98 | Down |
| FBXO32 | 0.00000416 | -2.4 | Down |
| C1orf21 | 0.00000419 | -1.71 | Down |
| KCMF1 | 0.0000042 | -1.56 | Down |
| MBP | 0.00000425 | -2.42 | Down |
| TRIM52-AS1 | 0.00000432 | -1.59 | Down |
| UBE2QL1 | 0.00000452 | -2.39 | Down |
| EGLN1 | 0.00000467 | -2.13 | Down |
| CPTP | 0.00000497 | -1.57 | Down |
| RGS9BP | 0.00000507 | -2.48 | Down |
| PKN1 | 0.00000513 | -1.67 | Down |
| DDX54 | 0.00000514 | -1.57 | Down |
| VWA8 | 0.0000052 | -1.71 | Down |
| DMTN | 0.00000539 | -2.5 | Down |
| KLHL34 | 0.00000555 | -3.84 | Down |
| COMTD1 | 0.00000555 | -1.76 | Down |
| KIAA1161 | 0.00000565 | -2.03 | Down |
| LSM14B | 0.00000566 | -1.53 | Down |
| ADAMTSL5 | 0.0000067 | -2.14 | Down |
| ZNF853 | 0.00000726 | -1.63 | Down |
| WIPF3 | 0.00000731 | -2.51 | Down |
| ST3GAL3 | 0.00000732 | -1.79 | Down |
| USP38 | 0.00000814 | -1.58 | Down |
| HYAL4 | 0.0000082 | -2.88 | Down |
| CRLF1 | 0.0000082 | -2.09 | Down |
| PPM1J | 0.00000841 | -1.86 | Down |
| SLC41A1 | 0.00000845 | -2.13 | Down |
| MAPK12 | 0.00000882 | -1.72 | Down |
| ALDH1L1 | 0.0000089 | -2.13 | Down |
| RXRG | 0.00000928 | -3.28 | Down |
| HINT3 | 0.00000948 | -2.32 | Down |
| ARNT2 | 0.00000948 | -1.69 | Down |
| ASB10 | 0.00000954 | -2.34 | Down |
| RCAN2 | 0.00000966 | -1.78 | Down |
| CEP85 | 0.00000973 | -1.58 | Down |
| ASB11 | 0.00000986 | -3.54 | Down |
| PNPO | 0.0000101 | -1.62 | Down |
| TPPP | 0.0000103 | -1.57 | Down |
| CHCHD10 | 0.0000104 | -1.63 | Down |
| ZNF787 | 0.0000107 | -1.78 | Down |
| HSPB6 | 0.0000111 | -2.69 | Down |
| USP2 | 0.0000115 | -3.37 | Down |
| CA14 | 0.0000118 | -2.46 | Down |
| ORAI1 | 0.0000118 | -1.99 | Down |
| CNKSR2 | 0.0000124 | -3.27 | Down |
| PKDCC | 0.0000125 | -2.04 | Down |
| NEURL1 | 0.0000126 | -2.37 | Down |
| OPTN | 0.0000135 | -1.53 | Down |
| SLC47A1 | 0.0000136 | -3.93 | Down |
| LINC01128 | 0.0000136 | -2.22 | Down |
| PTPN3 | 0.0000136 | -1.81 | Down |
| FXN | 0.0000142 | -1.83 | Down |
| TTN | 0.0000151 | -2.6 | Down |
| ST6GALNAC2 | 0.0000157 | -1.98 | Down |
| OSGIN1 | 0.0000158 | -1.7 | Down |
| ADCY9 | 0.000016 | -2.38 | Down |
| SMOC1 | 0.0000161 | -1.9 | Down |
| NR1D1///THRA | 0.0000165 | -3.26 | Down |
| VEGFA | 0.0000173 | -2.28 | Down |
| TRIP10 | 0.0000179 | -1.8 | Down |
| RNF128 | 0.000018 | -3.19 | Down |
| FOXP2 | 0.0000191 | -2.5 | Down |
| PLIN4 | 0.0000191 | -1.84 | Down |
| MACROD1 | 0.0000191 | -1.56 | Down |
| CNKSR1 | 0.0000192 | -1.57 | Down |
| COX5B | 0.0000193 | -1.75 | Down |
| HIST1H1C | 0.0000193 | -1.67 | Down |
| TMEM161B-AS1 | 0.0000197 | -1.81 | Down |
| RCL1 | 0.0000217 | -1.89 | Down |
| RASA4B///RASA4CP///RASA4 | 0.0000232 | -3.21 | Down |
| FKBP8 | 0.0000243 | -2.03 | Down |
| UNC13C | 0.0000283 | -3.41 | Down |
| RBM20 | 0.0000283 | -1.67 | Down |
| RPS11 | 0.0000291 | -2.15 | Down |
| FHL3 | 0.00003 | -2.02 | Down |
| CUEDC1 | 0.0000302 | -1.85 | Down |
| ST3GAL6 | 0.0000329 | -2.4 | Down |
| PITX3 | 0.0000329 | -2.01 | Down |
| MYOZ3 | 0.0000331 | -2.08 | Down |
| COQ8A | 0.0000347 | -2.28 | Down |
| NDUFV3 | 0.0000347 | -1.65 | Down |
| REPIN1 | 0.0000369 | -2.76 | Down |
| TMEM120A | 0.000037 | -1.96 | Down |
| TOM1 | 0.0000371 | -2.07 | Down |
| TRIM3 | 0.0000386 | -1.56 | Down |
| NINJ2 | 0.0000413 | -2.71 | Down |
| LINC00312 | 0.0000451 | -2.23 | Down |
| PPP1R1C | 0.0000454 | -1.66 | Down |
| HYAL1 | 0.0000469 | -2.4 | Down |
| GPD1L | 0.0000478 | -1.67 | Down |
| GRK2 | 0.0000501 | -1.81 | Down |
| RNF115 | 0.0000507 | -1.81 | Down |
| RAB11B | 0.0000567 | -2.17 | Down |
| NEK10 | 0.0000571 | -2.7 | Down |
| OBSCN | 0.0000571 | -1.99 | Down |
| NKAPL | 0.0000574 | -1.74 | Down |
| DENND2C | 0.0000593 | -2.14 | Down |
| KRT31 | 0.0000623 | -3.68 | Down |
| ZNF385B | 0.0000625 | -1.81 | Down |
| BEND7 | 0.0000645 | -1.54 | Down |
| CCR3 | 0.0000648 | -2.27 | Down |
| BHLHE41 | 0.0000654 | -2.12 | Down |
| PBX2 | 0.0000655 | -1.56 | Down |
| MYLK3 | 0.0000665 | -2.93 | Down |
| INPP5J | 0.0000665 | -1.84 | Down |
| WNK2 | 0.0000687 | -1.74 | Down |
| MYPOP | 0.0000692 | -1.72 | Down |
| LDHD | 0.0000697 | -1.86 | Down |
| MIR6883///PER1 | 0.0000701 | -2.51 | Down |
| UBTF | 0.0000702 | -1.78 | Down |
| MID1IP1 | 0.0000762 | -1.62 | Down |
| ALDH2 | 0.0000798 | -1.52 | Down |
| IKBKB | 0.0000824 | -1.59 | Down |
| HSPA2 | 0.0000838 | -1.95 | Down |
| KHDRBS3 | 0.0000861 | -1.68 | Down |
| PLEKHB1 | 0.0000886 | -1.74 | Down |
| DNM2 | 0.0000921 | -1.6 | Down |
| SOD2 | 0.0001 | -2.15 | Down |
| RPL27A | 0.000102 | -2.15 | Down |
| DKFZp779M0652 | 0.000106 | -1.76 | Down |
| INPP4B | 0.000111 | -1.84 | Down |
| SLC9A3R2 | 0.000114 | -1.92 | Down |
| STRN4 | 0.000117 | -1.57 | Down |
| SLC2A4 | 0.000118 | -2.47 | Down |
| RPL3L | 0.000118 | -1.78 | Down |
| SLC29A2 | 0.00012 | -1.89 | Down |
| PPARGC1A | 0.000126 | -1.51 | Down |
| TRIM63 | 0.000137 | -1.58 | Down |
| CEBPZOS | 0.00014 | -1.69 | Down |
| PKP2 | 0.000141 | -2.26 | Down |
| SLC6A10PB///SLC6A10P///SLC6A8 | 0.000144 | -1.91 | Down |
| CITED4 | 0.000144 | -1.85 | Down |
| MIR1-1HG | 0.000146 | -1.55 | Down |
| CIART | 0.00016 | -3.34 | Down |
| ATP2B2 | 0.000166 | -1.75 | Down |
| COQ9 | 0.000169 | -1.54 | Down |
| MAP2K6 | 0.000173 | -2.11 | Down |
| SLC22A23 | 0.000173 | -1.73 | Down |
| NEDD4 | 0.000175 | -1.62 | Down |
| RSPO3 | 0.000178 | -2.01 | Down |
| ENAM | 0.000184 | -1.81 | Down |
| IL12RB2 | 0.000185 | -2.69 | Down |
| SLC37A4 | 0.000194 | -1.85 | Down |
| PPARA | 0.000194 | -1.79 | Down |
| MTMR3 | 0.000195 | -1.7 | Down |
| IFNLR1 | 0.000209 | -1.86 | Down |
| ABCC1 | 0.000211 | -1.8 | Down |
| S100A1 | 0.000218 | -2.01 | Down |
| ENPP5 | 0.000221 | -2.05 | Down |
| PIR-FIGF///FIGF | 0.000231 | -1.54 | Down |
| SMAD7 | 0.000239 | -1.57 | Down |
| HOXA13 | 0.000248 | -1.77 | Down |
| VGLL2 | 0.000254 | -1.9 | Down |
| ARRDC2 | 0.000271 | -1.84 | Down |
| DMD | 0.000291 | -2.49 | Down |
| PRICKLE3 | 0.000296 | -1.9 | Down |
| SNAI3 | 0.000314 | -2.43 | Down |
| BDH1 | 0.00033 | -1.58 | Down |
| HBA2///HBA1 | 0.000332 | -2.3 | Down |
| PDE4D | 0.000338 | -1.56 | Down |
| GDNF | 0.000375 | -3.05 | Down |
| ZBED1 | 0.000378 | -1.63 | Down |
| SLC29A1 | 0.000383 | -1.59 | Down |
| P2RY2 | 0.000386 | -1.54 | Down |
| HOMER2 | 0.000405 | -1.62 | Down |
| SLC25A30 | 0.000418 | -1.74 | Down |
| ANKRD2 | 0.000426 | -2.28 | Down |
| SMCO1 | 0.000455 | -2.01 | Down |
| FHL2 | 0.000455 | -1.65 | Down |
| OPLAH | 0.000461 | -1.9 | Down |
| FGF11 | 0.000462 | -1.93 | Down |
| GREM1 | 0.000482 | -2.04 | Down |
| IDI2 | 0.000515 | -2.64 | Down |
| BSG | 0.000523 | -2.07 | Down |
| LSM12 | 0.000539 | -1.83 | Down |
| KIAA1217 | 0.000541 | -1.72 | Down |
| PROX1 | 0.000554 | -1.63 | Down |
| PDLIM3 | 0.000637 | -1.64 | Down |
| LMOD1 | 0.00067 | -2.08 | Down |
| MYADML2 | 0.000675 | -1.65 | Down |
| MYH11 | 0.000843 | -2.5 | Down |
| ANOS1 | 0.000843 | -1.51 | Down |
| LMO1 | 0.000977 | -1.74 | Down |
