# Supplementary material for: Identification of hub genes and therapeutic siRNAs to develop novel adjunctive therapy for Duchenne muscular dystrophy
Source: BMC Musculoskelet Disord. 2024 May 18;25:386. doi: 10.1186/s12891-024-07206-6 (PMC11102231; doi:10.1186/s12891-024-07206-6)
Supplement: Supplementary file 4 — Supplementary Material 4 [file 12891_2024_7206_MOESM4_ESM.docx]

**Supplementary table 4. The top 10 significant GO function of DEGs.**

| Expression | Category | GeneSet | Description | Enrichment  Ratio | pValue | FDR |
| --- | --- | --- | --- | --- | --- | --- |
| Up-regulation | BP | GO:0006955 | Immune response | 2.21 | 0 | 0 |
|  | BP | GO:0006952 | Defense response | 2.41 | 0 | 0 |
|  | BP | GO:0002682 | Regulation of immune system process | 2.19 | 0 | 0 |
|  | BP | GO:0045087 | Innate immune response | 2.71 | 0 | 0 |
|  | BP | GO:0006954 | Inflammatory response | 2.96 | 0 | 0 |
|  | BP | GO:0043062 | Extracellular structure organization | 3.65 | 0 | 0 |
|  | BP | GO:0030198 | Extracellular matrix organization | 3.74 | 0 | 0 |
|  | BP | GO:0016477 | Cell migration | 2.14 | 2.22E-16 | 2.52E-13 |
|  | BP | GO:0007155 | Cell adhesion | 2.12 | 4.44E-16 | 4.49E-13 |
|  | BP | GO:0002684 | Positive regulation of immune system process | 2.36 | 8.88E-16 | 7.34E-13 |
|  | CC | GO:0009986 | Cell surface | 2.96 | 0 | 0 |
|  | CC | GO:0031012 | Extracellular matrix | 5.02 | 0 | 0 |
|  | CC | GO:0062023 | Collagen-containing extracellular matrix | 6.24 | 0 | 0 |
|  | CC | GO:0005764 | Lysosome | 2.88 | 1.24E-14 | 3.13E-12 |
|  | CC | GO:0000323 | Lytic vacuole | 2.88 | 1.33E-14 | 3.13E-12 |
|  | CC | GO:0005773 | Vacuole | 2.73 | 1.61E-14 | 3.15E-12 |
|  | CC | GO:0044433 | Cytoplasmic vesicle part | 2.11 | 1.43E-13 | 2.40E-11 |
|  | CC | GO:0030141 | Secretory granule | 2.46 | 3.56E-12 | 5.22E-10 |
|  | CC | GO:0031226 | Intrinsic component of plasma membrane | 1.95 | 4.12E-12 | 5.38E-10 |
|  | CC | GO:0098805 | Whole membrane | 1.95 | 9.58E-12 | 1.13E-09 |
|  | MF | GO:0005201 | Extracellular matrix structural constituent | 6.63 | 0 | 0 |
|  | MF | GO:0044877 | Protein-containing complex binding | 2.17 | 2.09E-12 | 1.96E-09 |
|  | MF | GO:0005518 | Collagen binding | 7.25 | 5.77E-12 | 3.61E-09 |
|  | MF | GO:0005178 | Integrin binding | 4.43 | 3.96E-09 | 1.59E-06 |
|  | MF | GO:0043394 | Proteoglycan binding | 8.76 | 4.24E-09 | 1.59E-06 |
|  | MF | GO:0005198 | Structural molecule activity | 2.10 | 1.10E-08 | 3.43E-06 |
|  | MF | GO:0042802 | Identical protein binding | 1.66 | 5.70E-08 | 1.53E-05 |
|  | MF | GO:0030020 | Extracellular matrix structural constituent conferring tensile strength | 7.60 | 1.02E-07 | 2.39E-05 |
|  | MF | GO:0005539 | Glycosaminoglycan binding | 3.15 | 1.32E-07 | 2.74E-05 |
|  | MF | GO:0005102 | Signaling receptor binding | 1.66 | 2.29E-07 | 4.29E-05 |
| Down-regulation | BP | GO:0061061 | Muscle structure development | 3.40 | 7.82E-10 | 7.11E-06 |
|  | BP | GO:0006936 | Muscle contraction | 4.08 | 2.60E-08 | 1.03E-04 |
|  | BP | GO:0030029 | Actin filament-based process | 2.90 | 3.87E-08 | 1.03E-04 |
|  | BP | GO:1902305 | Regulation of sodium ion transmembrane transport | 10.14 | 4.54E-08 | 1.03E-04 |
|  | BP | GO:0003012 | Muscle system process | 3.57 | 7.68E-08 | 1.35E-04 |
|  | BP | GO:0002028 | Regulation of sodium ion transport | 8.23 | 8.90E-08 | 1.35E-04 |
|  | BP | GO:0042692 | Muscle cell differentiation | 3.91 | 1.15E-07 | 1.45E-04 |
|  | BP | GO:0060537 | Muscle tissue development | 3.73 | 1.28E-07 | 1.45E-04 |
|  | BP | GO:0043270 | Positive regulation of ion transport | 4.39 | 1.72E-07 | 1.74E-04 |
|  | BP | GO:0014706 | Striated muscle tissue development | 3.70 | 2.85E-07 | 2.59E-04 |
|  | CC | GO:0043292 | Contractile fiber | 6.61 | 2.85E-10 | 3.35E-07 |
|  | CC | GO:0044449 | Contractile fiber part | 6.65 | 8.14E-10 | 3.97E-07 |
|  | CC | GO:0030016 | Myofibril | 6.56 | 1.01E-09 | 3.97E-07 |
|  | CC | GO:0031674 | I band | 7.90 | 1.09E-08 | 3.02E-06 |
|  | CC | GO:0030017 | Sarcomere | 6.39 | 1.45E-08 | 3.02E-06 |
|  | CC | GO:0099512 | Supramolecular fiber | 2.99 | 1.67E-08 | 3.02E-06 |
|  | CC | GO:0099081 | Supramolecular polymer | 2.96 | 2.00E-08 | 3.02E-06 |
|  | CC | GO:0099080 | Supramolecular complex | 2.96 | 2.05E-08 | 3.02E-06 |
|  | CC | GO:0030018 | Z disc | 7.93 | 3.86E-08 | 5.04E-06 |
|  | CC | GO:0097458 | Neuron part | 2.15 | 8.05E-07 | 9.45E-05 |
|  | MF | GO:0008092 | Cytoskeletal protein binding | 2.66 | 2.11E-08 | 3.95E-05 |
|  | MF | GO:0003779 | Actin binding | 3.67 | 4.45E-08 | 4.18E-05 |
|  | MF | GO:0008307 | Structural constituent of muscle | 11.66 | 3.43E-07 | 2.15E-04 |
|  | MF | GO:0017080 | Sodium channel regulator activity | 12.82 | 9.60E-07 | 4.50E-04 |
|  | MF | GO:0005516 | Calmodulin binding | 4.34 | 1.03E-05 | 3.86E-03 |
