# Supplementary material for: Identification of hub genes and therapeutic siRNAs to develop novel adjunctive therapy for Duchenne muscular dystrophy
Source: BMC Musculoskelet Disord. 2024 May 18;25:386. doi: 10.1186/s12891-024-07206-6 (PMC11102231; doi:10.1186/s12891-024-07206-6)
Supplement: Supplementary file 5 — Supplementary Material 5 [file 12891_2024_7206_MOESM5_ESM.docx]

**Supplementary table 5. The list of genes in module 1 to 4**

| Module 1 | Module 2 | Module 3 | Module 4 |
| --- | --- | --- | --- |
| C3AR1 | GGH | SAMHD1 | CMTM6 |
| CCR1 | GBP3 | IFI44L | PLAU |
| CXCL12 | IRF7 | IFI44 | PLAUR |
| COL3A1 | PYCARD | IFIT3 | VAMP8 |
| APOE | TRIM38 | IFIT1 | LAIR1 |
| SDC2 | NPC2 | IFIT2 | LILRB2 |
| GNB4 | CTSC | MX2 | FCER1G |
| CCL5 | SP100 | HERC5 | ATP8B4 |
| CCR5 | HLA-DPB1 | RSAD2 | CD53 |
| COL5A3 | HLA-DPA1 | PSMB8 |  |
| IGFBP3 | HLA-DRA |  |  |
| ACKR3 | HLA-DRB1 |  |  |
| FN1 | TRIM22 |  |  |
| C3 | GBP1 |  |  |
| COL15A1 | MID1 |  |  |
| COL14A1 | IRF8 |  |  |
| PPIB | LYZ |  |  |
| FPR3 | ANXA2 |  |  |
| COL6A6 | CECR1 |  |  |
| LEPRE1 | TUBB |  |  |
| FSTL1 | VCAM1 |  |  |
| VCAN |  |  |  |
| LPAR1 |  |  |  |
| CHRDL1 |  |  |  |
| LAMB1 |  |  |  |
| FBN1 |  |  |  |
| TIMP1 |  |  |  |
| COL6A3 |  |  |  |
| CCR2 |  |  |  |
| COL21A1 |  |  |  |
| COL4A1 |  |  |  |
| SERPINH1 |  |  |  |
| ADRA2A |  |  |  |
| CXCR4 |  |  |  |
| COL5A1 |  |  |  |
| FAM20A |  |  |  |
| GOLM1 |  |  |  |
| GPC3 |  |  |  |
| COL5A2 |  |  |  |
| COL1A2 |  |  |  |
| ANXA1 |  |  |  |
| ADCY3 |  |  |  |
