# Supplementary figures and images for: Identification of hub genes and therapeutic siRNAs to develop novel adjunctive therapy for Duchenne muscular dystrophy
Source: BMC Musculoskelet Disord. 2024 May 18;25:386. doi: 10.1186/s12891-024-07206-6 (PMC11102231; doi:10.1186/s12891-024-07206-6)

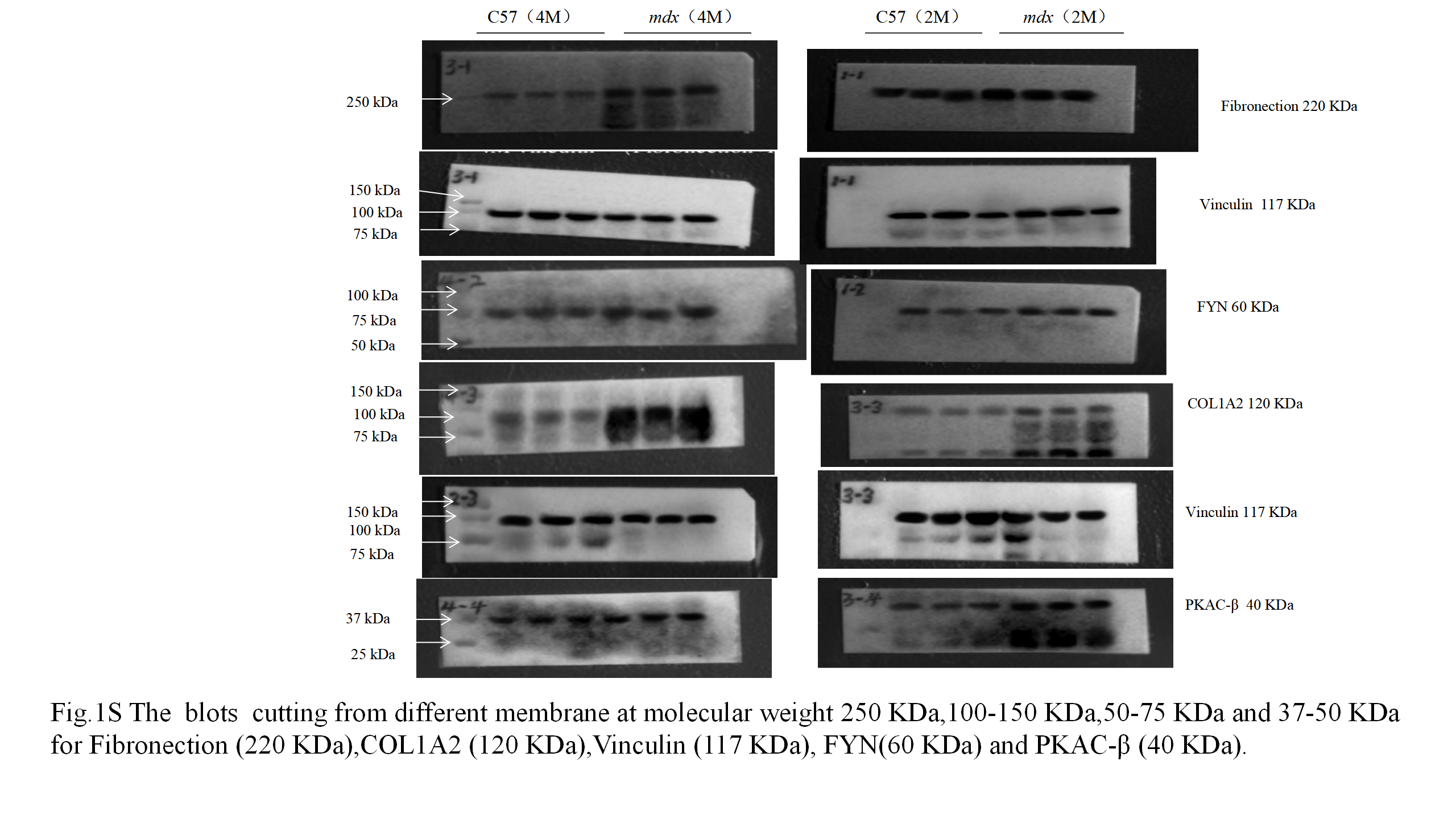

Supplement: Supplementary file 6 — Supplementary Material 6 [file 12891_2024_7206_MOESM6_ESM.png]
